# Inhibitory effect of natural flavone luteolin on *Streptococcus mutans* biofilm formation

**DOI:** 10.1128/spectrum.05223-22

**Published:** 2023-09-21

**Authors:** Lucille Rudin, Noelle Roth, Julien Kneubühler, Badri Nath Dubey, Michael M. Bornstein, Viktoriya Shyp

**Affiliations:** 1 Department Research, University Center for Dental Medicine Basel UZB, University of Basel, Basel, Switzerland; 2 CSSB Centre for Structural Systems Biology, Deutsches Elektronen-Synchrotron DESY, Notkestr, Hamburg, Germany; 3 Department of Oral Health and Medicine, University Center for Dental Medicine Basel UZB, University of Basel, Basel, Switzerland; The Ohio State University Division of Biosciences, Columbus, Ohio, USA

**Keywords:** flavonoids, dental caries, polysaccharides, bacteria, microscopy, amyloidogenic proteins

## Abstract

*Streptococcus mutans* is one of the key pathogens responsible for dental caries, which is known to be one of the most prevalent biofilm-associated diseases worldwide. *S. mutans* virulence strongly depends on its biofilm formation and enamel demineralization abilities due to the production of surface adhesins, exopolysaccharides, and acid in the presence of sugar. Luteolin is an abundant natural flavone with a prominent anti-bacterial function. However, it remains unclear how luteolin affects *S. mutans* pathogenicity including its acidogenicity and biofilm formation. In this study, the effect of luteolin on *S. mutans* growth, acid production, and its early and late biofilm formation and biofilm disruption was tested. Luteolin shows strong anti-biofilm activity, while it remains non-toxic for bacterial cell viability. In the biofilm, luteolin reduces the expression of *S. mutans* virulence genes such as *gbpC*, *spaP*, *gtfBCD*, and *ftf* encoding for surface adhesins and extracellular polysaccharides (EPS)-producing enzymes, which reflects in the strong reduction of bacteria and EPS. Further, it reduces water-insoluble glucan production in the biofilm, potentially, via direct interference with glucosyltransfereases (Gtfs). Moreover, at biofilm inhibitory concentrations, luteolin significantly reduces acid production by *S. mutans*. Finally, luteolin could target *S. mutans* amyloid proteins to disrupt the biofilm based on the observation that it inhibits the uptake of the amyloid dye, thioflavin T, by *S. mutans* extracellular proteins and failed to inhibit biofilm formation by the mutant strain lacking three main amyloid proteins. In conclusion, luteolin appears to be a potent natural compound with pleiotropic anti-biofilm properties against one of the main cariogenic human pathogens, *S. mutans*.

**IMPORTANCE:**

Flavonoids are natural compounds with proven anti-bacterial and anti-biofilm properties. Here, we describe the anti-biofilm properties of natural flavone luteolin against the main cariogenic bacteria, *S. mutans*. Luteolin inhibited gene expression of cell surface adhesins, fructosyltransferases, and glucosyltransferases, which promotes a significant reduction of bacterial and EPS biomass in early and late biofilms. Moreover, luteolin could directly target *S. mutans* Gtfs and functional amyloids to modulate pathogenic biofilms. These observations provide important insights into the anti-biofilm properties of luteolin while laying out a framework for future therapeutic strategies targeting biofilm-associated virulence factors of oral pathogens.

## INTRODUCTION

Dental caries is one of the most widespread biofilm-associated oral infectious diseases worldwide that can significantly impact quality of life and is associated with high economic cost (1). Pathogenic potential of the oral biofilm depends on the prevalence and physiological functions of *Streptococcus mutans*, a major etiological agent of dental caries. *S. mutans* encodes multiple virulence factors to establish and persist within the oral biofilm (2 – 4). Thus, it produces several cell surface adhesins such as glucan-binding proteins (5) and SpaP (6, 7), which promote direct bacterial interaction with the surfaces and other components of the biofilm, hence contributing to the initial colonization and polymicrobial biofilm formation. Furthermore, *S. mutans* expresses several glucosyltransferases (Gtfs) (8, 9) and fructosyltransferase (10) essential for the production of extracellular polysaccharides (EPS) such as glucan and fructan, respectively (11, 12). Moreover, Gtfs are essential components of the salivary pellicle formed on the tooth enamel or hydroxyapatite (HA) surfaces where they produce glucan and provide binding sites for the components of the polymicrobial oral biofilm. EPS matrix production by *S. mutans* plays a crucial role in dental biofilm structure, stability, and resistance to anti-bacterial treatment (13, 14). Regularly exposed to the dietary sucrose, *S. mutans* liberates lactic acid, which makes its biofilm a direct cause of tooth surface demineralization (2 – 4). Additionally, recent findings revealed that *S. mutans* produces several functional amyloid proteins, such as SpaP, WapA, and Smu_63c, which contribute to the stabilization of the biofilm (15, 16).

**FIG 1:**
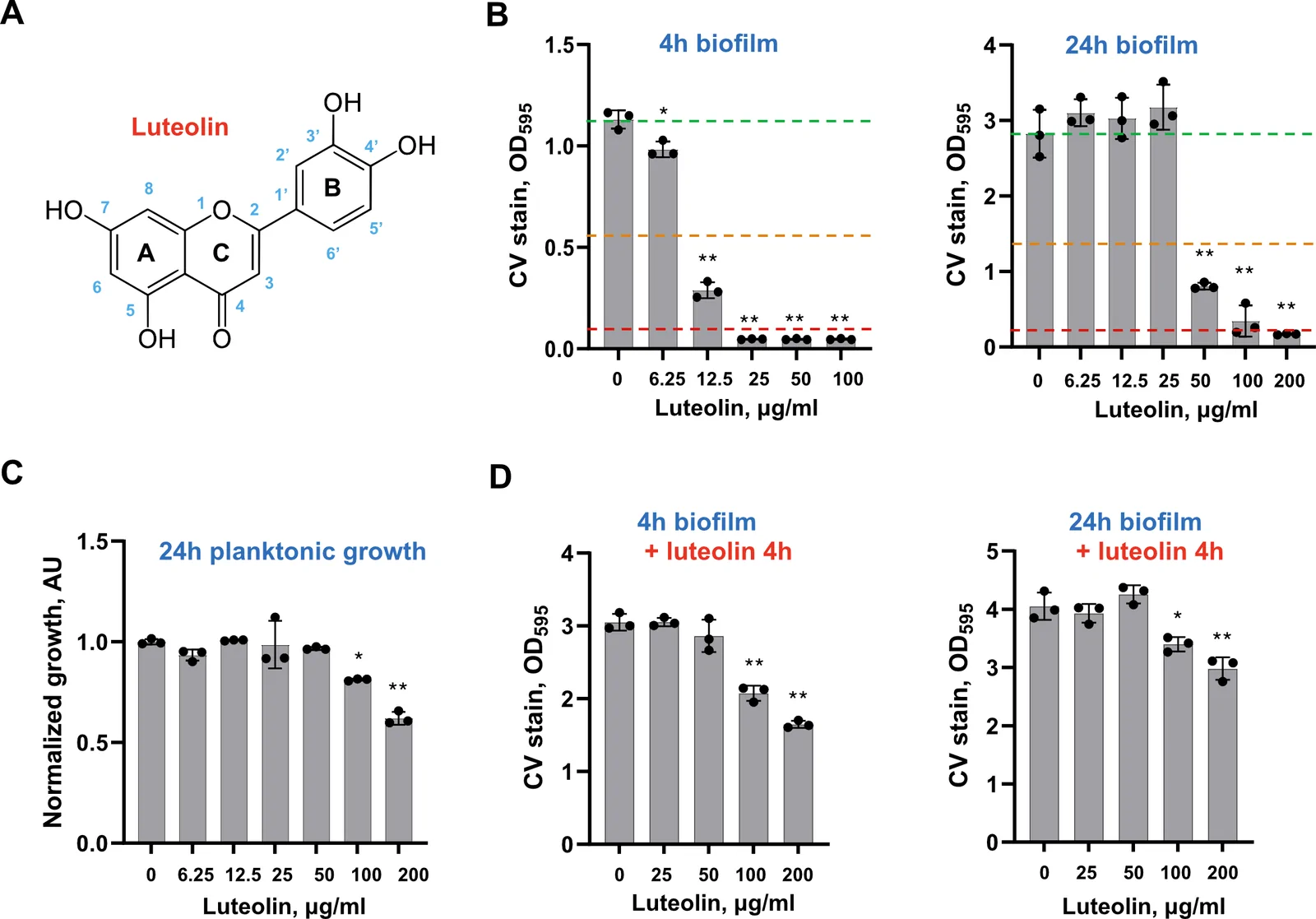
Anti-biofilm effect of luteolin on *S. mutans* early and mature biofilm formation. (**A**) Chemical structure of luteolin. (**B**) The effect of luteolin on early (4-h) and late (24-h) biofilms of *S. mutans*. Biofilm mass was quantified by crystal violet staining (CV stain) and measuring absorbance at 595 nm. (**C**) Planktonic growth of *S. mutans* strain in BHI supplemented with luteolin. Bacterial growth was monitored by measuring optical density at 600 nm. (**D**) The effect of luteolin on preformed 4- and 24-h biofilm. In all panels, bars represent the mean of three biological replicates. Error bars show standard deviation. Green dashed line indicates the value of the control experiment without luteolin. Orange and red dashed lines indicate the reduction in biofilm mass up to 50% and 90%, respectively. Bars represent the mean of three biological replicates. Error bars show standard deviation. *P < 0.05, **P < 0.0001.

Since conventional anti-cariogenic therapies are non-selective and often associated with side effects and risk of resistance development, therapeutic strategies targeting biofilm-associated virulence factors of oral pathogens provide an attractive alternative. Recently, plant polyphenols and flavonoids, which represent a significant part of human diet, have gained a growing interest as anti-microbial alternatives with potent anti-biofilm properties (17 – 19). Herbal extracts with a mixture of flavonoids, as well as pure compounds, exhibited anti-microbial activity on different bacterial and fungal pathogens including oral streptococci, *Escherichia coli*, *Staphylococcus aureus*, *Bacillus subtilis*, and *Candida albicans*. (20 – 23). The dietary flavone apigenin (4′,5,7-trihydoxyflavone), an abundant component found in propolis and various fruits and vegetables, displays a strong inhibitory effect against *S. mutans* biofilm via interference with the physiology, metabolism, and virulence gene expression of the bacterium (24, 25). Combining apigenin with sodium fluoride was highly effective in caries prevention (24). Structurally similar flavone luteolin (3′,4′,5,7-tetrahydoxyflavone) (Fig. 1A), occurring in various herbs, fruits, and vegetables (26, 27), possesses a variety of pharmacological activities, including anti-oxidant, anti-inflammatory, and anti-carcinogenic. Recent studies revealed that luteolin and herbal extracts containing luteolin possess anti-bacterial and anti-biofilm activity against various oral pathogens in single and mixed biofilms (20, 28). However, the effect of luteolin on the main cariogenic pathogen, *S. mutans*, remains elusive. Hence, the purpose of this study was to investigate the anti-bacterial and anti-biofilm effects of luteolin on *S. mutans* and to preliminarily reveal the possible underlying mechanisms, which could provide the basis for a future use of luteolin in oral therapeutic agents such as rinses or toothpastes.

## RESULTS

### Luteolin inhibits *S. mutans* biofilm formation with minor effect on bacterial growth

Twofold serial dilutions of luteolin were used to investigate its effect on *S. mutans* early attachment—within the first 4 h of biofilm formation—as well as on mature biofilm grown for 24 h. As shown in Fig. 1B, at concentrations above 12.5 µg/mL, luteolin strongly inhibits biofilm formation within the first 4 h upon surface attachment (>75% of biofilm inhibition), while higher concentrations, i.e., above 50 µg/mL, are needed to inhibit 24-h biofilm correspondingly. Remarkably, at biofilm inhibitory concentrations, luteolin had no significant effect on *S. mutans* growth and cell viability in planktonic culture and in biofilm (Fig. 1C; Fig. S1 and S2). Luteolin has further been tested to eradicate preformed *S. mutans* biofilm, since it has been recently shown to effectively disperse established single- and dual-species biofilms of *C. albicans* and *Enterococcus faecalis*. The application of luteolin on early and late preformed biofilms of *S. mutans* revealed that at a concentration above 100 µg/mL, luteolin can partially decrease an early 4-h biofilm but has noticeably less potential to disassemble a mature 24-h biofilm (Fig. 1D). To further investigate whether this effect is due to biofilm dispersal, the density of bacteria released to the fresh medium after luteolin treatment was monitored. At concentrations above 50 µg/mL, luteolin significantly decreased bacterial cell detachment from the preformed 4-h biofilm, hence inhibiting the dispersal (Fig. S3A). Similarly, at concentrations above 100 µg/mL, luteolin reduced bacterial dispersal from the 24-h preformed biofilm (Fig. S3B). CLSM analysis of the 4-h preformed biofilm treated with solvent only revealed more attached single cells, chains, and monolayered biofilm spread on the surface. In contrast, treatment with 50 and 100 µg/mL of luteolin gradually decreased the amount of single-cell colonizers, resulting in a few attached isolated micro- and macrocolonies (Fig. S3C).

**FIG 2:**
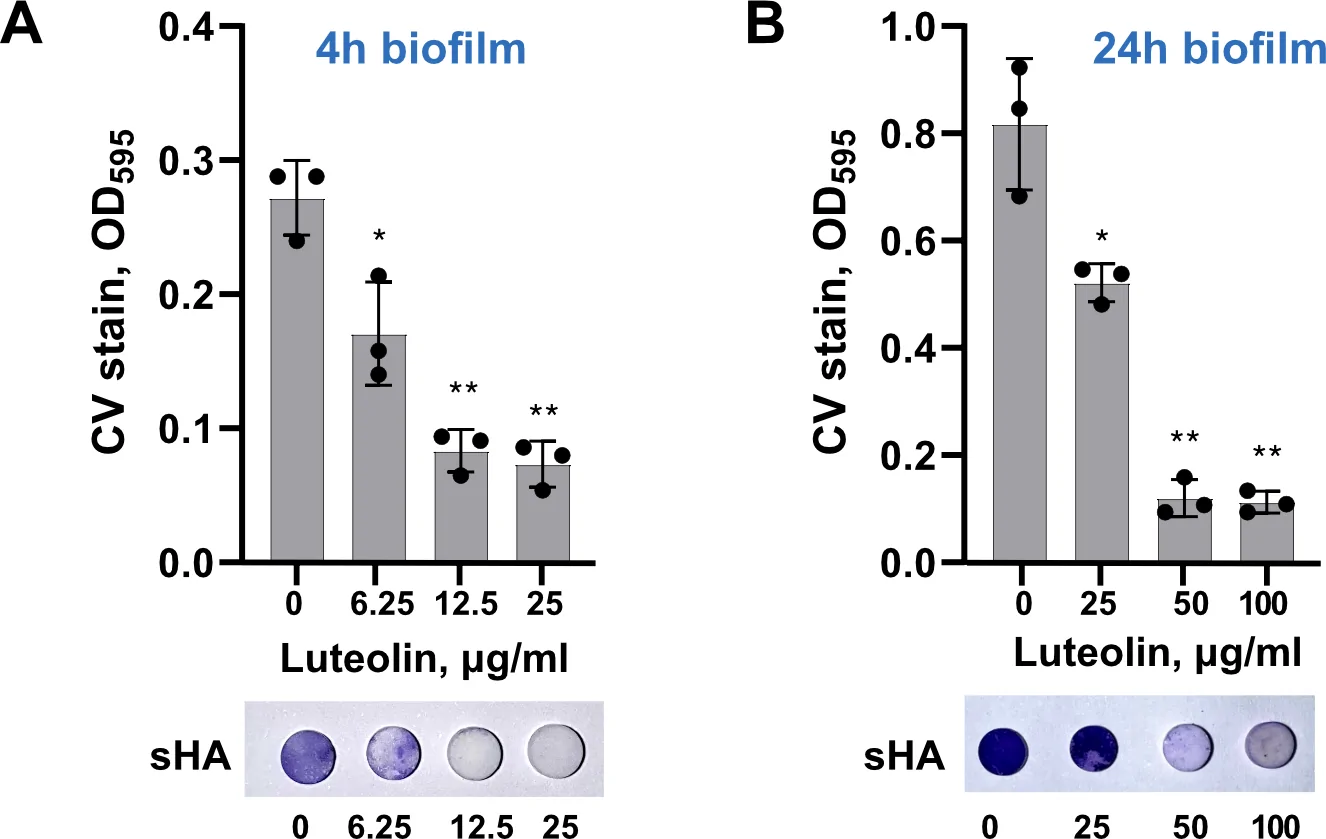
Luteolin effect on *S. mutans* adherence to HA. Upper panel: the effect of luteolin on early, 4-h (**A**), and late, 24-h (**B**), biofilms of *S. mutans* formed on sHA. Lower panel: representative sHA disks stained with crystal violet. Biofilms were quantified by crystal violet staining and measuring absorbance at 595 nm. In all panels, bars represent the mean of three biological replicates. Error bars show standard deviation. . *P < 0.05, **P < 0.0001.

Next, to mimic the biofilm formation condition in human oral environment, luteolin was tested on *S. mutans* biofilm formed on saliva-coated hydroxyapatite (sHA) surfaces. Bacterial adhesion was evaluated by crystal violet staining after 4 and 24 h, similar to conditions described above. As shown in Fig. 2, *S. mutans* adhesion to sHA was dramatically inhibited by 12.5 µg/mL and 50.0 µg/mL of luteolin in 4 and 24 h, respectively. Notably, its anti-biofilm activity turned out to be very similar to apigenin, a well-known structurally similar flavone representative with anti-biofilm properties against *S. mutans*. In contrast, isoflavone 7,3′,4′-trihydroxyisoflavone (THIF), wich shares similarity to luteolin hydroxylation of the B ring, does not exhibit effective anti-biofilm activity (Fig. S4).

### Quantitative analysis of bacteria and EPS in luteolin-treated biofilms

**FIG 3:**
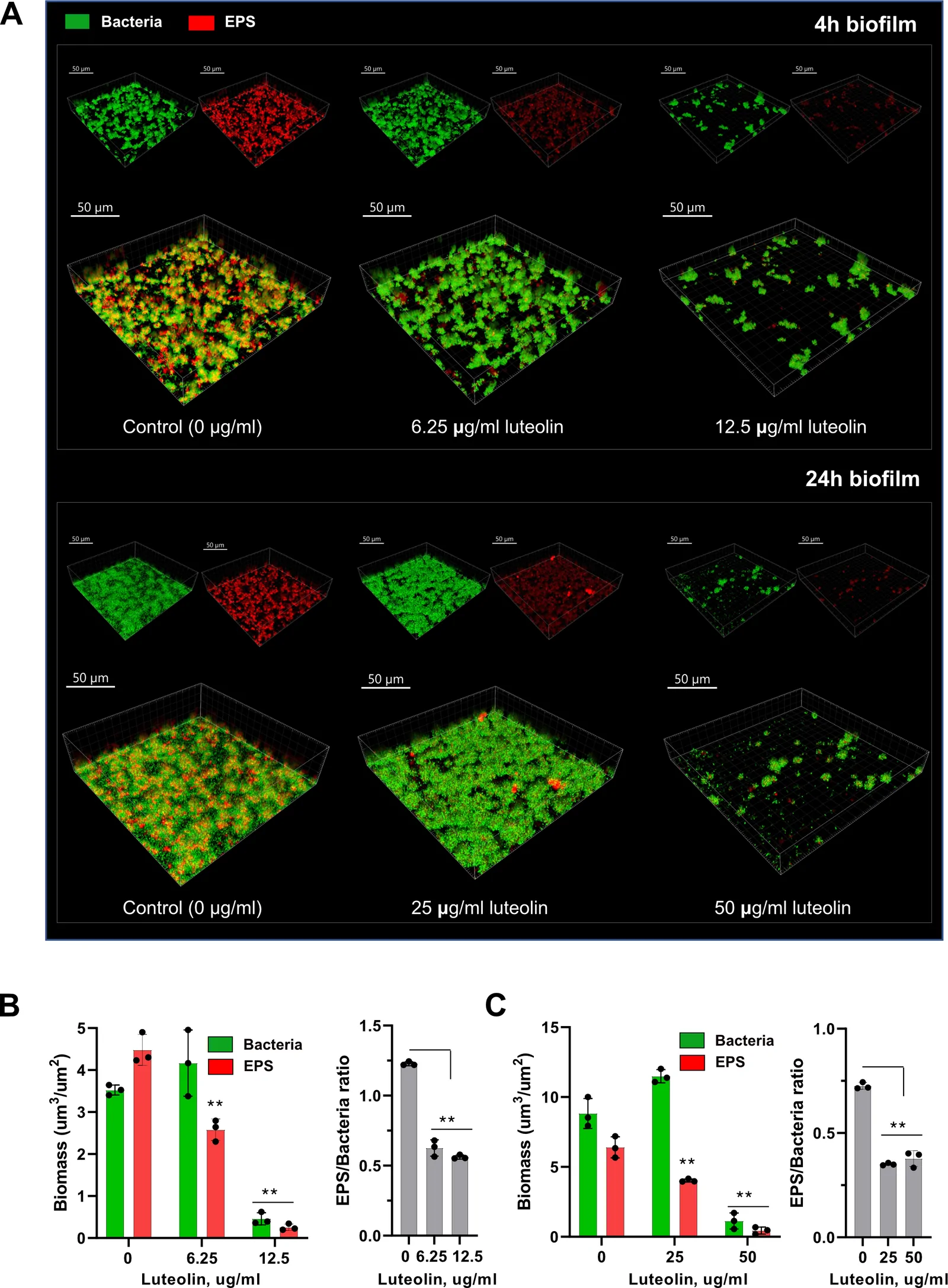
Biofilm structure and morphology affected by luteolin. (**A**) Representative images of the double-labeled 4- and 24-h biofilms. Bacterial cells are shown in green and EPS are in red. Three-dimensional reconstructions were performed with Imaris version 9.0.0. (**B and C**) Bacterial and EPS biomass (left panel) and EPS:bacteria ratio as calculated by Imaris for 4-h biofilm (**B**) and 24-h biofilm (**C**). Bars represent the mean of three randomly selected positions per sample. Error bars show standard deviation. **P < 0.0001

To evaluate the inhibitory effect of luteolin on biofilm structure and the biomass of bacteria and EPS, confocal laser scanning microscopy (CLSM) was used combined with a double staining, where bacteria were labeled with green fluorescent SYTO 9 and EPS were stained with red-labeled fluorescent dextran. For the efficient image analysis, the biofilms were cultured in an 8-well chambered borosilicate coverglass system for 4 and 24 h. In accordance with the crystal violet staining assay (Fig. 1B), on a borosilicate coverglass, less luteolin is needed to vastly reduce 4-h biofilm (above 12.5 µg/mL), while mature biofilm required concentrations above 50 µg/mL of luteolin (Fig. 3A). Three-dimensional (3D) biofilm images analysis indicated that, at all tested concentrations, luteolin gradually decreased EPS biomass in comparison to the control and, subsequently, notably reduced EPS:bacteria ratio in each tested condition (Fig. 3B and C).

### Luteolin reduces biofilm-associated virulence gene expression of *S. mutans* and affects glucan production

**FIG 4:**
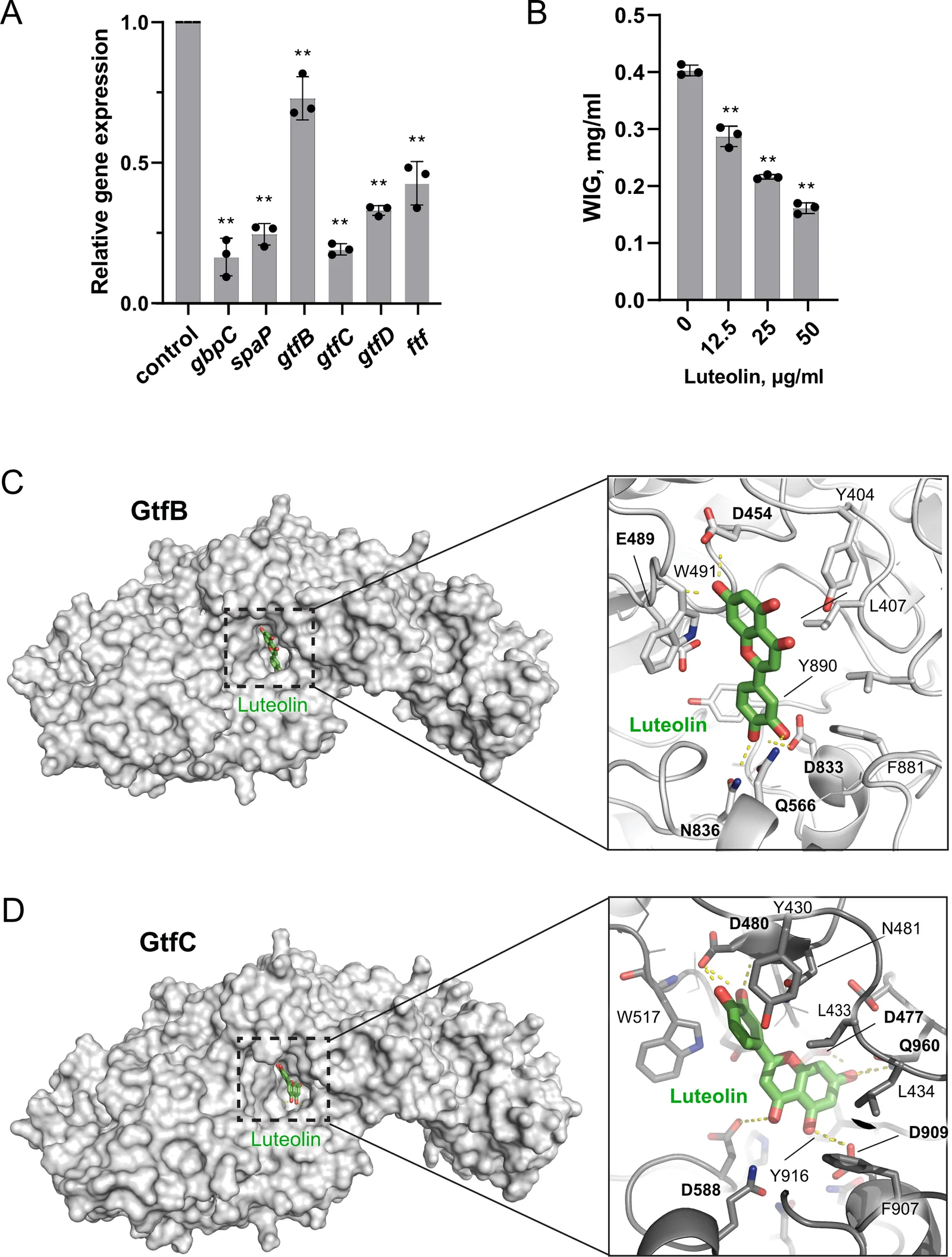
Inhibitory effect of luteolin on gene expression and glucan production in *S. mutans* biofilm. (**A**) Inhibitory effect of luteolin on the biofilm-associated gene expression levels in 24-h biofilm. The relative expression levels were quantified by real-time PCR with 16S rRNA as an internal control. (**B**) Luteolin effect on biofilm-associated water-insoluble glucans as measured by anthrone method. (**C**) Left: surface representation of luteolin (shown in green stick) docked with GtfB (PDB code 8FK4). Right: best-docked pose of luteolin in the active sites of *S. mutans* GtfB. (**D**) Left: surface representation of luteolin docked with GtfC (PDB code 3AIE). Right: best-docked pose of luteolin in the active sites of *S. mutans.* GtfC residues involved in hydrogen bonding are labeled as bold. In all panels, bars represent the mean of three biological replicate. Error bars show standard deviation. **P < 0.0001.

To further investigate the effect of luteolin on *S. mutans* biofilm, gene expression levels of the key effectors regulating bacterial surface adhesion and EPS formation were analyzed. To this end, biofilms were treated with 25 μg/mL of luteolin for 24 h; total RNA was extracted from the biofilms; and relative mRNA levels were determined for *spaP* and *gbpC*, as well as *gtfBCD* and *ftf*. The results (Fig. 4A) revealed a reduction in transcriptional levels of all tested genes in comparison to the untreated biofilm, with the strongest effect on *spaP*, *gbpC*, and *gtfC* genes expression levels.

To assess the effect of luteolin on EPS synthesis by glucosyltransferases, water-insoluble glucan (WIG) accumulation in biofilms was analyzed by anthrone-sulfuric methods. WIG was extracted from the 24-h biofilms treated with 12.5, 25.0, and 50.0 µg/mL of luteolin. As shown in Fig. 4B, with increasing luteolin concentration, WIG production was inhibited to approximately >29%, >45%, and >54% in comparison to the untreated control biofilm. To further investigate the inhibitory potential of luteolin on *S. mutans* Gtfs, molecular docking studies were performed. To this end, the interactions between luteolin and two glucosyltransferases contributing to the WIG synthesis, GtfB and GtfC, were studied. Fig. 4C and D shows the best-docked pose of luteolin in the active sites of GtfB and GtfC, respectively. Luteolin formed hydrogen bonds with multiple conserved amino acids in the subsite −1 of GtfC including Asp480, Asp909, Glu960, and, particularly, Asp477 and Asp588, responsible for the binding and stabilizing of glucosyl moiety of the sucrose during transglycosylation reaction (Fig. 4D; Fig. S5B). Similarly, luteolin interacted with several conserved amino acids in the active site of GtfB (Fig. 4C, Fig. S5A) including Glu489 and Asp883 involved in Gtfs inhibitor acarbose binding. Luteolin was found to interact with GtfB and GtfC with a binding energy of −7.31 and −7.4 kcal/mol, respectively. In comparison, apigenin, a known inhibitor of Gtfs, formed similar however slightly weaker interactions in the GtfB and GtfC active sites with a binding energy of −6.63 and −6.84 kcal/mol, respectively (Fig. S6). Consequently, WIG production was inhibited to approximately >23% in the biofilm treated with 50 µg/mL of apigenin (Fig. S7).

### Luteolin inhibits acid production by *S. mutans*

**FIG 5:**
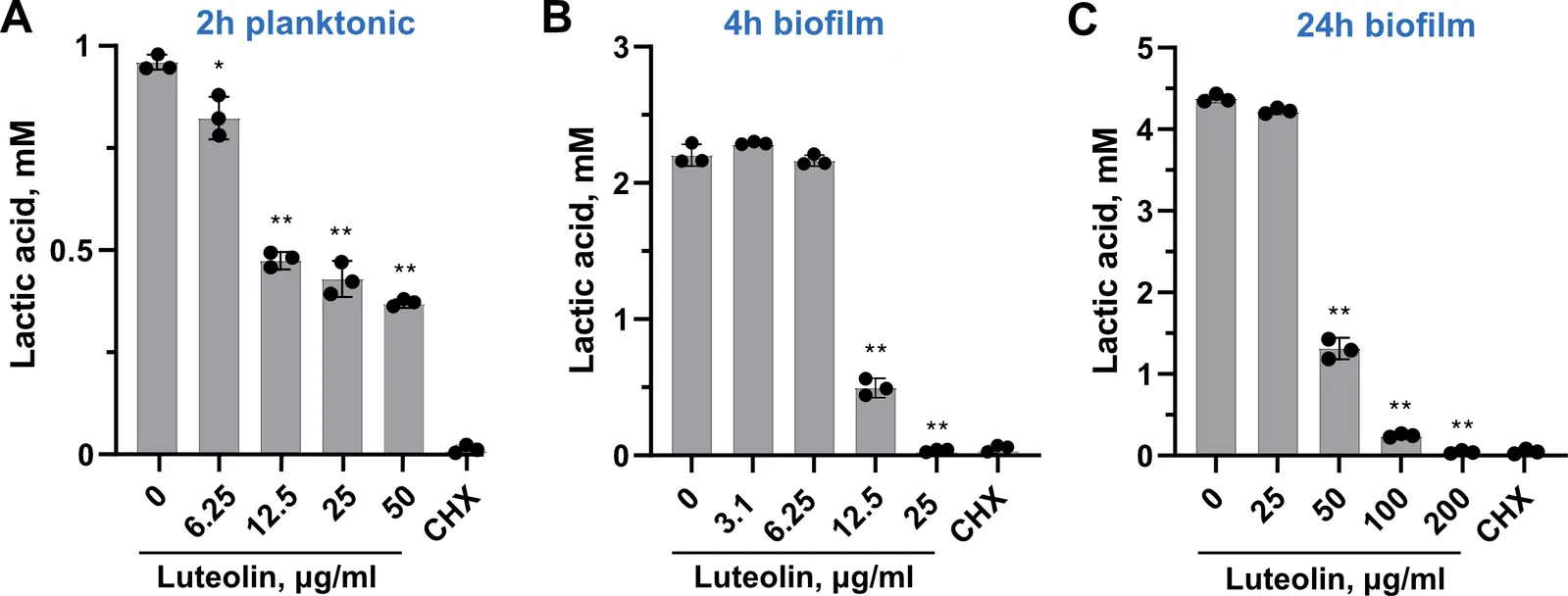
Inhibitory effect of luteolin on lactic acid production by *S. mutans*. (**A**) Lactic acid measured in planktonic culture of *S. mutans* treated with luteolin for 2 h. (**B and C**) Lactic acid measured in treated 4-h (**B**) and 24-h (**C**) biofilms of *S. mutans*. Bars represent the mean of three biological replicates. Error bars show standard deviation. CHX stand for cultures treated with 0.01% chlorhexidine and served as a negative control. *P < 0.05, **P < 0.0001.

To assess the effect of luteolin on *S. mutans* acidogenicity, lactic acid production in bacterial culture and biofilm treated with luteolin was monitored. As shown in Fig. 5A, 2-h treatment of the planktonic culture of the *S. mutans* with luteolin at the concentration above 12.5 μg/mL markedly inhibited the lactic acid production compared to the untreated control. In early and late biofilms treated with luteolin, decrease of lactic acid production correlated with decreased bacterial biomass (Fig. 5B and C).

### Luteolin interferes with *S. mutans* extracellular amyloid proteins to disrupt the biofilm

**FIG 6:**
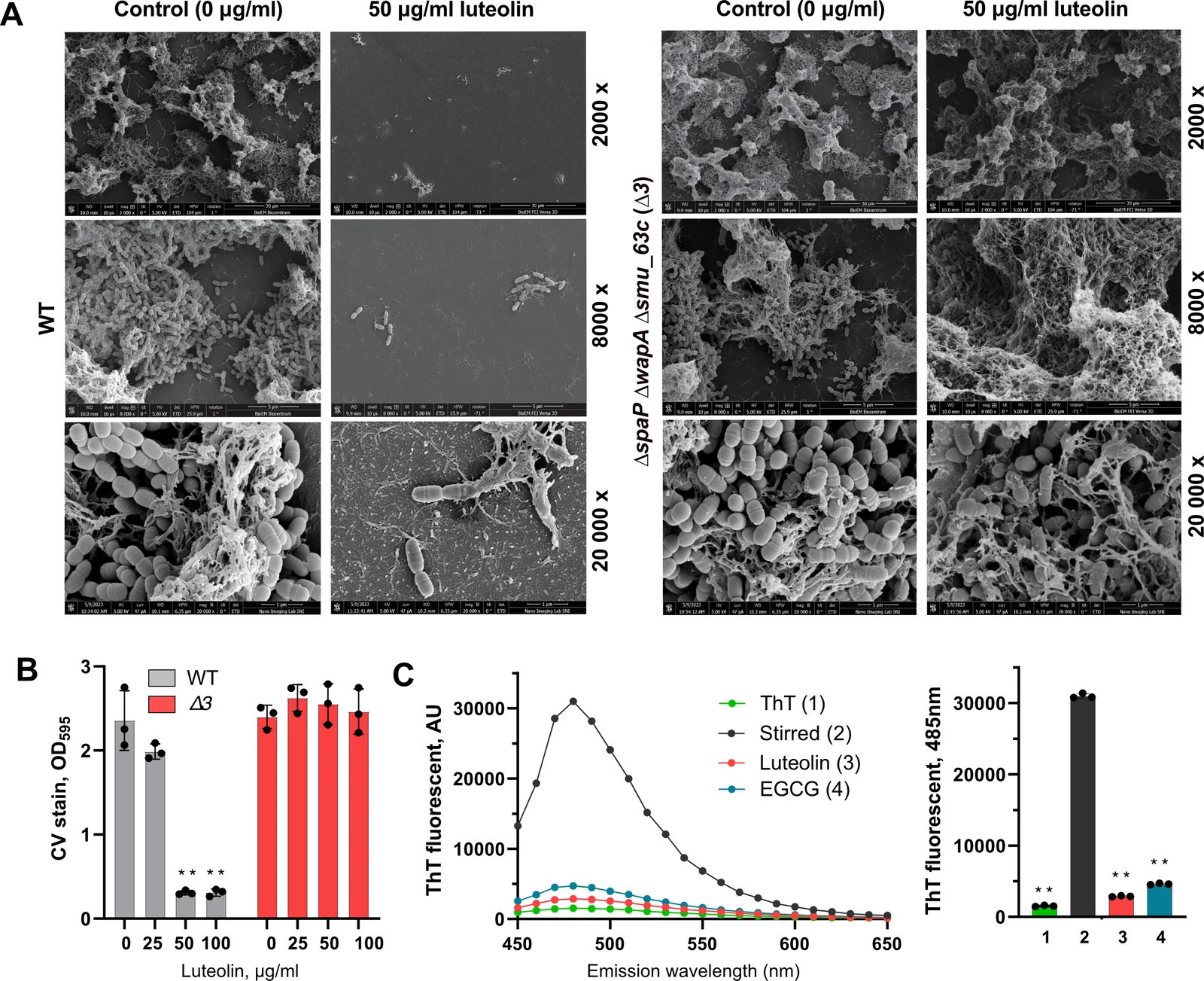
Anti-amyloidogenic activity of luteolin in *S. mutans*. (**A**) Representative scanning electron microscopy images of *S. mutans* wild type (WT) and *Δ3* mutant biofilm architecture treated with 50 μg/mL of luteolin compared to non-treated control. Images were taken at ×2,000, ×8,000, and ×20,000 magnification. (**B**) The effect of luteolin on 24-h biofilms of *S. mutans* wild type (in gray) and *Δ3* mutant (in red) as quantified by crystal violet staining. Bars represent the mean of three biological replicates. Error bars show standard deviation. (**C**) ThT fluorescence emission spectra (left) and pick fluorescence (right) following excitation at 442 nm. *S. mutans* extracellular proteins were stirred in the cold to induce amyloid fibrillization in the absence of inhibitors (black), in the presence of luteolin (red) or EGCG (blue). ThT background fluorescence was evaluated in phosphate-buffered saline (green). **P < 0.0001.

In addition to extracellular polysaccharides, *S. mutans* produces several amyloidogenic proteins such as SpaP, WapA, and Smu_063c, which contribute to the development and architecture of the biofilm matrix. Since luteolin has a strong and direct anti-amyloidogenic activity in *E. coli*, in this study, it has been further tested whether luteolin interferes with *S. mutans* amyloid proteins as components of the biofilm matrix. To this end, a triple-deletion mutant lacking *spaP*, *wapA*, and *smu_063*c genes (*Δ3*) was constructed and tested for 24-h biofilm inhibition assay. Crystal violet staining assay as well as scanning electron microscopy demonstrated that, at biofilm inhibitory concentrations above 50 µg/mL, luteolin does not affect *Δ3* mutant strain biofilm formation (Fig. 6A and B). These results suggest that *S. mutans* amyloid proteins could serve as a target for biofilm inhibition by luteolin. To further investigate whether luteolin interferes with amyloid formation by *S. mutans*, its extracellular protein fraction has been tested for thioflavin T (ThT)-dependent fluorescence, a specific indicator of amyloid fibrillization. To this end, to induce fibrillization, extracellular protein fraction has been stirred in the cold for 60 h alone or in the presence of 50 µg/mL of luteolin. Additionally, a well-known amyloid inhibitor, epigallocatechin-3-gallate (EGCG), has been used as a control. Following the excitation at 442 nm, the fluorescence intensity of ThT significantly increased for the stirred sample without inhibitors, while both luteolin and EGCG reduced ThT fluorescence (Fig. 6C), suggesting their similar, anti-amyloidogenic activity. Notably, apigenin is equally effective against both wild type and *Δ3* mutant strains, suggesting no interference of this flavone with amyloidogenic proteins of *S. mutans* (Fig. S8).

### Luteolin does not exhibit anti-bacterial effect against commensal streptococcal species

**FIG 7:**
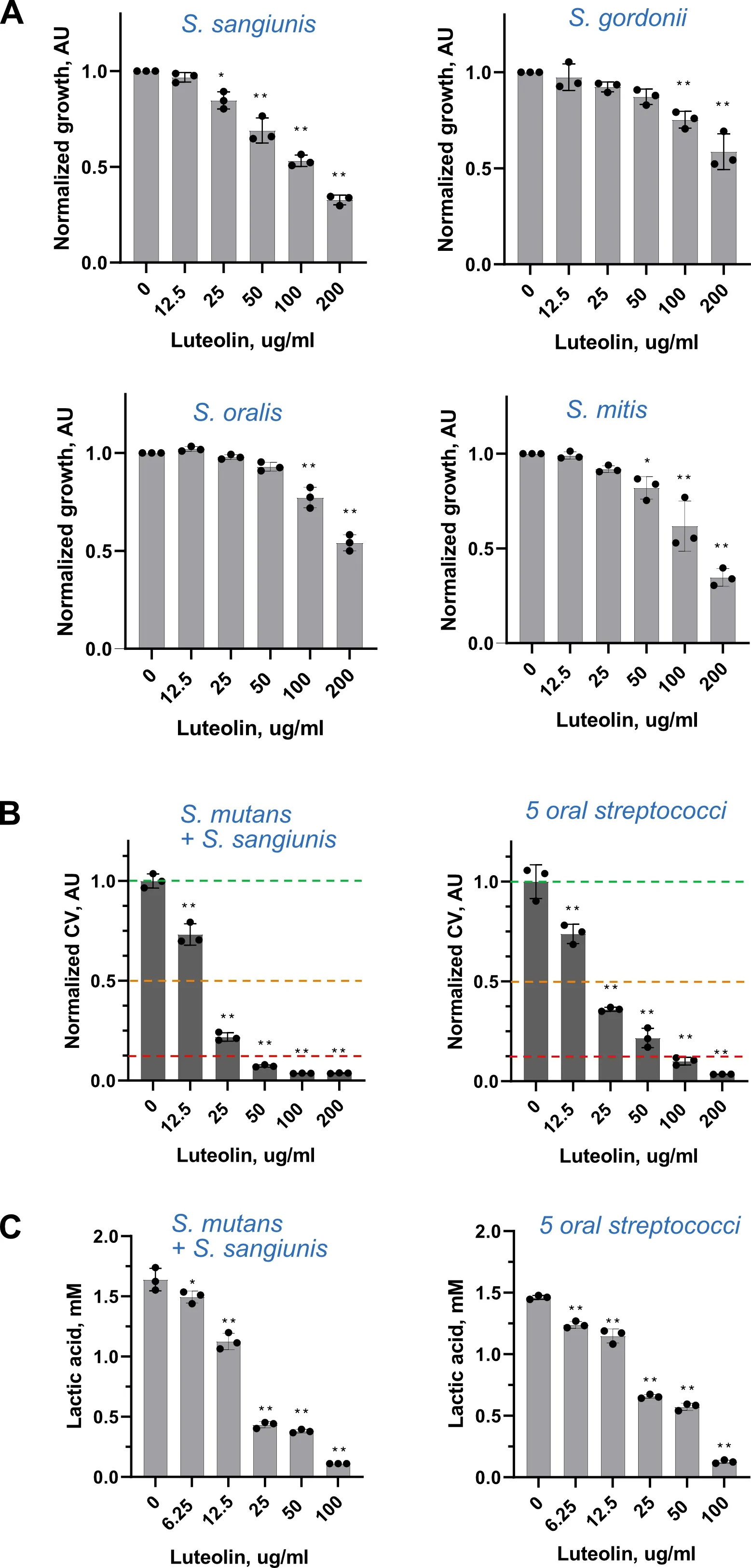
Luteolin effect on oral commensal streptococcal species. (**A**) Planktonic growth of indicated commensal streptococcal species in BHI supplemented with luteolin. Bacterial growth was monitored by measuring optical density at 600 nm and normalized to the untreated control. (**B**) The effect of luteolin on *S. mutan-S. sanguinis* dual-species and multi-species biofilm. Green dashed line indicates the value of the control experiment without luteolin. Orange and red dashed lines indicate the reduction in biofilm mass up to 50% and 90%, respectively. (**C**) Lactic acid produced by *S. mutan-S. sanguinis* dual-species and multi-species biofilm after treatment with luteolin. In all panels, bars represent the mean of three biological replicates. Error bars show standard deviation. *P < 0.05, **P < 0.0001.

To assess the effect of luteolin on oral commensal streptococci, planktonic growth of *Streptococcus sanguinis*, *Streptococcus gordonii*, *Streptococcus oralis*, and *Streptococcus mitis* have been evaluated in the presence of flavonoid at concentrations ranging from 12.5 to 200 μg/mL. The growth of all tested streptococcal species was not affected by luteolin at 12.5 µg/mL, the concentration used to inhibit early biofilm of *S. mutans*. At 50 µg/mL, the concentration used to inhibit 24-h biofilm, luteolin reduced the growth of *S. sanguinis*, *S. gordonii*, *S. oralis*, and *S. mitis* by 31%, 12.7%, 7.1%, and 18.1%, respectively. None of the tested luteolin concentrations exhibited bacteriostatic or bactericidal effect on oral commensal bacteria (Fig. 7A). To investigate the impact of luteolin on oral streptococci biofilm formation, 24-h biofilm assay was conducted for heterotypic biofilm models. While luteolin had similar impact on *S. mutans*-*S. gordonii*, *S. mutans*-*S. oralis*, and *S. mutans*-*S. mitis* dual-species biofilms as compared to the single-species *S. mutans* biofilm (Fig. S9), it was more effective to inhibit *S. mutans*-*S. sanguinis* dual-species biofilm and multi-species biofilm with five oral streptococcal strains (Fig. 7B). Thus, 12 and 25 µg/mL of luteolin effectively reduced *S. mutans*-*S. sanguinis* dual species by 30.7% and 83.7%, respectively, and reduced five-species biofilm by 26.2% and 63.0%, respectively. Consequently, these biofilms produced significantly less amount of lactic acid in comparison to the monospecies *S. mutans* biofilm (Fig. 7C).

### Luteolin biocompatibility to human oral fibroblasts

**FIG 8:**
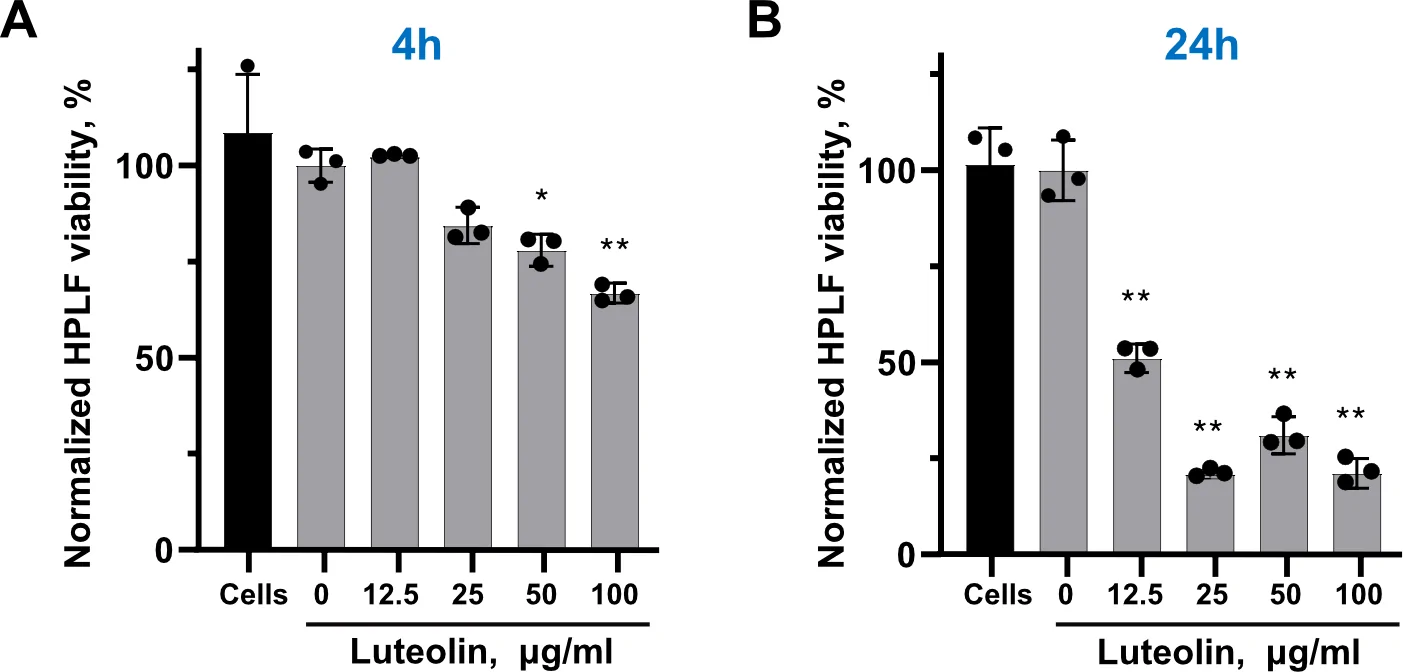
Cytotoxic effect of luteolin. Metabolic activity of HPLF cells after 4 h (**A**) and 24 h (**B**) of incubation with luteolin. Black bars indicate non-treated HPLF cell. Cells treated with 3% DMSO (0 µg/ml of luteolin) were used as control. In all panels, bars represent the mean of three biological replicates. Error bars show standard deviation. *P < 0.05, **P < 0.0001.

To investigate the cytotoxicity of luteolin, we treated human periodontal ligament fibroblasts (HPLFs) cells with the luteolin concentrations used in the *S. mutans* biofilm assay. HPLF cultured without luteolin as well as cells treated with 3% dimethyl sulfoxide (DMSO) were used as controls. MTS-based measurements of cell viability after 4 h of incubation with luteolin (Fig. 8A) show that at concentrations below 25 μg/mL, luteolin does not affect cell viability, while higher concentrations significantly reduce viability (down to 70% viability). After 24 h of incubation, the viability of HPLF cells decreased down to 50% for 12.5 μg/mL of luteolin and down to 20% for higher, biofilm-inhibitory concentrations (Fig. 8B).

## DISCUSSION

Pathologic changes in oral microbiota are the major driver of dental caries, the most prevalent biofilm-associated disease worldwide of the oral cavity (1). Conventional anti-cariogenic therapies rely on non-specific anti-bacterial agents, such as chlorhexidine digluconate or fluoride. However, they suppress resident flora and are often associated with side effects and risk of resistance development. To enhance the effectiveness of existing anti-cariogenic therapies, its combination with additional cariostatic agents, specifically targeting the virulence of the pathogenic members of the oral microbiome, provides an attractive alternative. *S. mutans* is a key component of pathogenic dental biofilm and is a primary etiological agent of dental caries. Its surface colonization and the ability to develop cariogenic biofilm depend on multiple virulence factors. They include synthesis and secretion of the surface adhesins and glucan-binding proteins as well as extracellular biofilm matrix production containing exopolysaccharides and amyloid fibrils (3, 14, 29). In this study, natural flavone luteolin has been tested against biofilm-associated physiological aspects of *S. mutans*.

First, luteolin exerts a strong dose-dependent inhibition of *S. mutans* biofilm formation with better performance on initial attachment then on 24-h biofilms. Thus, lower luteolin concentrations are needed to significantly reduce *S. mutans* biofilm formation within the first 4 h of surface attachment. Notably, this inhibitory concentration exhibits good biocompatibility to the human periodontal ligament fibroblast cells in contrast to the concentrations needed for 24-h biofilm inhibition. Such a strong effect on human oral cell viability could be explained by the fact that luteolin affects multiple cellular mechanisms, including induction of apoptotic cell death and suppression of cellular kinases and cell cycle (27). Therefore, finding an optimal condition for both strong anti-biofilm activity and low cytotoxicity is essential for oral cavity health and safety upon anti-cariogenic treatment.

Similar to another flavone apigenin, luteolin does not affect the planktonic growth and viability of *S. mutans* at the concentrations used to markedly reduce early and late biofilm, suggesting its primarily biofilm disintegrating activity rather than being anti-microbial against this bacterium. Intriguingly, while it is toxic for various pathogenic microbes such as *Enterococcus faecalis*, *Escherichia coli*, *Staphylococcus aureus*, and *Candida albicans* (20, 30), in its biofilm inhibitory concentrations, luteolin does not kill commensal streptococcal species such as *S. sanguinis*, *S. oralis*, *S. gordonii*, and *S. mitis* (Fig. 7), the first colonizers of the tooth surfaces and the main antagonists of *S. mutans*. Moreover, the increased susceptibility of dual- and mixed-species biofilm of *S. mutans* with oral commensals to luteolin treatment makes it a prominent non-toxic candidate to prevent dental plaque formation and development. However, more studies of luteolin on multi-species dental biofilms are needed to elucidate its selectivity against *S. mutans*.

The initial surface adhesion of *S. mutans* is the most important step in dental biofilm development and strongly depends on the bacterial surface properties and abilities to interact with biofilm components. Thus, surface protein antigens, mainly SpaP (31), and a major cell surface glucan-binding protein GbpC modulate *S. mutans* cell hydrophobicity and surface adhesion (32). Luteolin shows an inhibitory effect on the expression levels of *spaP* and *gbpC* genes, which could explain its prominent anti-biofilm activity, particularly on early biofilm formation. Interestingly, in contrast to its biofilm dispersal activity described for *C. albicans* and *E. faecalis* (20), in *S. mutans*, luteolin seemed to prevent bacterial cell detachment from the preformed biofilm, hence inhibiting colonization of the new surfaces (Fig. S3).

Further, though, to a different extent, luteolin reduces gene expression levels of all glycosyltransferases and fructosyltransferase, responsible for the synthesis of soluble and insoluble glucan and fructan, respectively. The suppression of indicated virulence factors impacts the structure of the biofilm and might contribute to the reduction of bacteria and EPS biomass in early and late biofilms observed with CLSM. Although these effects resemble the activity of other flavonoids including structurally similar apigenin, the exact mechanism of gene expression regulations by flavonoids remains yet unknown. Many flavonoids are identified as inhibitors of Gtfs in solution and on sHA surface. Their structural characteristics, namely the amount and position of hydroxyl groups and the presence of a C2-C3 double bond may provide sites for nucleophilic-based enzyme inhibition (33). In current study, 3′,4′,5,7-tetrahydoxyflavone luteolin, which also possesses a C2-C3 double bond, was shown to effectively inhibit *S. mutans* biofilm formation on sHA surfaces, suggesting that it could be another effective inhibitor of Gtfs under conditions similar to those of the oral environment. Molecular docking studies revealed that luteolin, similar to apigenin, interacts with crucial amino acid residues in Gtfs active site forming hydrogen bonding and hydrophobic and Van der Waals interactions (Fig. 4 C and D; Fig. S6). These interactions could interfere with the transglycosylation activity of Gtfs, leading to the inhibition of glucan synthesis in the biofilm (Fig. 4B; Fig. S7). In contrast, reduced hydroxylation at the critical position of the A ring (C5 position) in THIF as well as its altered spatial geometry, presumably, compromised its biofilm inhibitory potential (Fig. S7).

The apparent structure-activity relationship of flavonoids (22), namely, the presence of two free hydroxyl groups in the B ring of luteolin, also makes it a promising agent with anti-amyloidogenic properties. Upon biofilm development and maturation, amyloidogenic proteins of *S. mutans* contribute to the biofilm structural integrity and architecture. Plant polyphenols and flavonoids such as tannic acids and EGCG exhibited anti-biofilm activity against *S. mutans* via reduction of amyloid fibril formation (15, 16). Similarly, in our study, the *S. mutans* strain lacking three main amyloid proteins, SpaP, WapA, and Smu_063c, shows significant resistance to the biofilm inhibitory concentrations of luteolin, suggesting these proteins to be the potential targets for luteolin. Moreover, similar to well-known amyloid inhibitor EGCG, luteolin inhibited extracellular amyloid fibrillization based on ThT uptake assay. However, further studies of a direct effect of luteolin on amyloid protein oligomerization *in vitro* are needed to dissect the detailed mechanism of this inhibition.

Finally, one of the most essential virulence characteristics of the *S. mutans* is its ability to effectively produce lactic acid from the dietary sugars. Acidic environment, in turn, promotes the pathogenic changes in dental biofilms, which subsequently lead to tooth demineralization and dental caries. *S. mutans* monospecies early and late biofilms as well as mixed biofilms inhibited by luteolin appeared to be less acidogenic, while luteolin also repressed lactic acid production by *S. mutans* planktonic cells, hence extending its physiological targets and functional repertoire as an anti-cariogenic compound. This could give luteolin an additional advantage over other flavonoids with anti-biofilm properties as well as over single-target anti-bacterial agents. Further studies are worth performing to investigate a combinational potential of luteolin with other flavonoids and currently available anti-cariogenic agents such as fluoride or CHX to potentially introduce it to a toothpaste or mouthrinse.

## MATERIALS AND METHODS

### Bacterial strains and growth condition

All bacterial strains were purchased from the Leibniz Institute DSMZ-German Collection of Microorganisms and Cell Cultures. *Streptococcus mutans* ATCC 25175 (DSM 20523), *Streptococcus sanguinis* ATCC 10556 (DSM 20567), *Streptococcus gordonii* ATCC10558 (DSM 6777), *Streptococcus mitis* ATCC 49456 (DSM 12643), and *Streptococcus oralis* ATCC35037 (DSM 20627) were used in this study. Flavonoids were purchased from Sigma-Aldrich or Abcam, dissolved in 100% dimethyl sulfoxide (DMSO, Sigma-Aldrich, Switzerland) to a stock concentration 20 mg/mL, aliquoted and stored at −20°C. The bacteria were routinely cultured in brain heart infusion (BHI) broth (Oxoid, Thermo Scientific) aerobically at 37°C in 5% CO_2_. For the planktonic growth assay, fresh *S. mutans* culture (OD_600_ = 0.5–0.6) was diluted 1:100 in a 96-well plate using BHI and, if indicated, serial dilutions of flavonoids in DMSO. BHI supplemented with DMSO only was used a control. Final concentration of DMSO did not exceed 3%. Bacterial growth was monitored by measuring optical density at 600 nm by multi-mode plate-reader (Synergy HTX, BioTek, Agilent Technologies, Basel, Switzerland). For the biofilm assay, BHI was additionally supplemented with 1% (wt/vol) sucrose (BHIS). A triple-deletion mutant (*Δ3*) lacking P1 (SpaP), WapA, and Smu_63c amyloidogenic proteins (16) was generated in the same *S. mutans* background using a markerless mutagenesis strategy described previously (34, 35) (see supplemental materials for details).

### Biofilm inhibition assay

Crystal violet staining assay was used to evaluate the inhibitory effect of flavonoids on biofilm. A fresh *S. mutans* culture (OD_600_ = 0.5–0.6) was diluted 1:100 in 96-well plate using BHIS and, if indicated, serial dilutions of flavonoids in DMSO. BHIS supplemented with DMSO was used a control. Final concentration of DMSO did not exceed 3%. Bacterial suspension was incubated aerobically at 37°C. After 4 h or 24 h of incubation, excess medium including the planktonic cells was removed; the plate was washed twice with sterile water and air-dried for 20 min. An equal volume of 0.1% (wt/vol) crystal violet solution was added to each well and incubated for 10 min at room temperature. The crystal violet solution was then removed and the plate was washed with water. To dissolve the dye, an equal volume of 25% acetic acid was added to each well, and absorbance at 595 nm was measured by a plate reader (Synergy HTX, BioTek, Agilent Technologies).

For biofilm assay on hydroxyapatite, hydroxyapatite disks (5-mm diameter; HiMed Inc, USA) were coated with filter-sterilized clarified human saliva at room temperature for 20 min (sHA), and placed to the wells in a 96-well plate containing bacteria in BHIS with luteolin dilutions. Culture plate was incubated for 4 h and 24 h as described above. After incubation, sHA disks were washed with water to remove loosely attached cells and stained with crystal violet.

### Biofilm disruption assay

The 4- and 24-h biofilms of *S. mutans* formed in a 96-well plate in BHIS. Liquid medium containing planktonic and loosely attached cell was removed, and the biofilms were thoroughly washed with the sterile phosphate-buffered saline (PBS). Equal volume of the fresh BHI containing luteolin at a concentration range from 25 to 200 μg/mL was added to the wells and incubated for another 4 h at 37°C aerobically. After incubation, excess medium was removed; the plate was washed twice with sterile water, air-dried, and stained as described above. To asses biofilm dispersal, optical density of the removed excess medium with detached cell was monitored. For CLSM images of the preformed biofilm treated with luteolin, 4-h biofilm treated with 50 and 100 µg/mL on the eight-well chambered borosilicate coverglass plate was used. Biofilm treated with BHI supplemented with solvent (3% DMSO) was used as a control.

### Biofilm analysis and structural imaging

For the biofilm analysis by confocal laser scanning microscopy, the culture wild-type *S. mutans* was prepared as described above, and 0.5 mL was added to the eight-well chambered borosilicate coverglass plate. Plates were incubated aerobically for 4 h or 24 h. The bacterial cells and EPS were labeled with SYTO 9 (Invitrogen) and Alexa Fluor 647-labeled dextran conjugate (Thermo Fisher Scientific, USA), respectively, according to the manufacturer’s instruction. The biofilms were visualized on Leica SP8 confocal laser scanning microscope (Leica Microsystem, Switzerland) using ×63 oil immersion objective. The images were collected using 500/618- and 650/778-nm spectra for SYTO9 and Alexa Fluor 647, respectively. Each biofilm was scanned at three randomly selected positions. Three-dimensional reconstruction and analysis of the biofilms were performed with Imaris version 9.0.0 (Bitplane, Zürich, Switzerland).

For biofilm architecture analysis by scanning electron microscopy, the cultures of either wild-type or mutant streptococci were prepared as described above, and 1 mL was added to the 12-well plate containing glass coverslip. After 24 h of incubation, the biofilms were washed three times with sterile PBS to remove planktonic and loosely adherent cells and then fixed with 4% paraformaldehyde for 1 h at 4°C. The coverslips were washed once with PBS, dehydrated in ethanol series, and submersed in hexamethyldissilazane according to the method described previously (36). The samples were coated with platinum gold alloy before being analyzed on FEI Versa 3D scanning electron microscope (Thermo Fisher Scientific).

### Transcriptional analysis of biofilm-associated virulence factors

Total RNA isolation and purification from *S. mutans* biofilms and cDNA synthesis were performed as previously described with modifications (see supplemental materials for details). To quantify relative mRNA levels of *spaP*, *gpbC*, *gftBCD*, and *ftf* genes, real-time PCR was used, with 16S rRNA as an internal control. 2^−ΔΔCt^ method was used to analyze the data.

### Water-insoluble glucan measurement

The effect of 12.5, 25.0, and 50.0 µg/mL of luteolin on water-insoluble glucans was estimated by the anthrone-sulfuric methods as described previously (37) with modifications. Briefly, a fresh *S. mutans* culture (OD_600_ = 0.5–0.6) was diluted 1:100 in 24-well plates with BHIS containing luteolin. Biofilm was formed for 24 h at 37°C. After incubation, the culture medium was removed and the biofilm was carefully rinsed with sterile PBS. One milliliter of sterile PBS was added to each well, and the biofilm was scraped from the bottom of the well with a sterile spatula. The suspension was centrifuged at 6,000 *g* for 10 min, and WIG was extracted from the cell sediment in 4 mL of 0.4 M NaOH with constant agitation for 2 h at 37°C. After incubation, cells were collected by centrifugation (6,000 *g*, 10 min). Supernatant from alkaline extraction was mixed with three volumes of anthrone-sulfuric acid reagent and heated at 95°C for 6 min. The absorbance at 625 nm was measured in a 96-well plate on a microplate reader (Synergy HTX, BioTek, Agilent Technologies). The concentration of WIG was calculated according to the standard curve prepared with the glucose standards. The experiment was performed in triplicates (independent bacterial cultures). Biofilm formed in BHIS with 5% DMSO was used a negative control.

### *In silico* docking analysis

3D chemical structures of both luteolin and apigenin were obtained and energy minimized using Avogadro (version 1.2.0). The crystal structure of GtfC (PDB id: 3AIE) and GtfB (PDB id: 8FK4) was retrieved from the Research Collaboratory for Structural Bioinformatics (RCSB) PDB. While pre-processing the modeled GtfC and GtfB as receptors for docking, all the heteroatoms were removed. AutoDock (version 4.2) (38) suit incorporated in MGL tools (version 1.5.6) was used to perform molecular docking. For setting up the docking site, the grid (size = 100 × 70 × 60 points in XYZ dimensions) was mapped to the active site with default spacing (i.e., 0.375 Å) and grid potential was evaluated, followed by 100 genetic algorithm (GA) runs of molecular docking. Clustering approach was used to select the best pose from most populated cluster. All visualizations were made using Pymol (version 2.0 ed, Schrödinger, LLC). LIGPLOT+ (version 2.2) (39) was used to identify molecular interactions within the protein-ligand complex.

### Lactic acid measurement

The effect of luteolin on *S. mutans* lactic acid production was evaluated for its biofilm inhibitory concentrations. A fresh *S. mutans* culture (OD_600_ = 0.5–0.6) was harvested and washed twice with PBS. Cells were resuspended 1:10 in buffered peptone water (BPW) supplemented with 0.2% sucrose in a 96-well plate. Twofold serial dilutions of luteolin in DMSO were added to the wells. Untreated bacterial cells were used as a negative control; 0.1% chlorhexidine was used as a positive control. Bacterial suspension was incubated aerobically at 37°C for 2 h. *S. mutans* cells were removed by centrifugation (8,000 *g*, 5 min, 4°C), and the supernatant was used to measure lactic acid concentration with a lactate assay kit (MAK064, Sigma-Aldrich) according to the manufacturer’s instruction. The measurements were performed with three biological replicates.

For lactic acid measurements in biofilm, 4- and 24-h *S*. *mutans* biofilms treated with luteolin were formed in a 96-well plate as described above. After incubation, excess media were removed and biofilms were washed two times with the sterile PBS. An equal volume of BPW supplemented with 0.2% sucrose was added to each well and incubated aerobically at 37°C for 2 h. Supernatant was used to measure lactic acid as described above.

### ThT assay

Inhibition of *S. mutans* extracellular amyloid fibrillization by luteolin was tested by measuring the ThT fluorescence spectrum. For this, *S. mutans* strain ATCC 25175 was grown in a biofilm medium supplemented with 0.8% glucose (40) and cultured overnight at 37°C. After incubation, cells were removed by centrifugation at 7,000 *g* for 15 min. The supernatant containing extracellular proteins was filtered through a 0.2 μm Nalgene filter, concentrated with 10-kDa-cutoff Amicon centrifuge filters (Millipore) and equilibrated in PBS buffer, pH 7.2. Aliquots of extracellular proteins were stirred at 4°C for 60 h with 50 μg/mL luteolin. Following stirring, ThT was added to the samples to the final concentration of 2 μM, mixed, and incubated in the dark for 30 min at room temperature. Next, 200 μL of samples was transferred to a black-walled 96-well plate, excited at 442 nm, and fluorescent emission spectra were measured from 450 to 650 nm in a Synergy H1 hybrid reader (BioTek). A protein sample stirred in the absence of luteolin served as the positive control. A ThT-only sample was used as the negative control.

### Biocompatibility

The cytotoxicity of luteolin on human periodontal ligament fibroblast cells was evaluated by the 3-(4,5-dimethylthiazol-2-yl)-5-(3-carboxymethoxyphenyl)-2-(4-sulfophenyl)-2H-tetrazolium (MTS) assay following instructions from the manufacturer (CellTiter 96 AQueous One Solution Cell Proliferation Assay, Promega, USA). HPLF cell line and all media and reagents were purchased from ScienCell (Chemie Brunschwig AG, Basel, Switzerland). The cells were cultured in Fibroblast Medium supplemented with 2% FBS, 1% growth factors, and 1% penicillin-streptomycin (FM+ medium) in a humidified atmosphere at 37°C in 5% CO_2_. After three passages, cells were cultured in 96-well plates (3,000 cells per well) alone, with 3% DMSO or with luteolin in its biofilm inhibitory concentrations. After 4 h and 24 h of incubation, the cells were washed, and 200 µL FM+ medium with 20 µL CellTiter 96 Aqueous One Solution Reagent was added to each well and the plates were incubated at 37°C in 5% CO_2_ for 2 h. The absorbance was measured at 490 nm using a microplate reader (Synergy HTX, BioTek, Agilent Technologies). Cells cultured with DMSO and without luteolin served as control.

### Statistical analysis

All assays were performed in triplicate. The results are presented as mean ± standard deviation. Statistically significant differences were determined with one-way analysis of variance followed by Dunnett’s multiple comparisons test: *P < 0.05, **P < 0.0001. The results were graphed using GraphPad Prism version 9.0.2 (GraphPad Software, LLC, USA).

## SUPPLEMENTAL MATERIAL

Supplemental figures spectrum.05223-22-s0001.docx Fig. S1 to S9.

Tables S1 and S2 spectrum.05223-22-s0002.pdf Primers used for mutant strain construction and for qRT-PCR.

Supplemental methods spectrum.05223-22-s0003.pdf Additional experimental details for *S. mutans* mutant strain construction, trasformation, and real-time PCR.

ASM does not own the copyrights to Supplemental Material that may be linked to, or accessed through, an article. The authors have granted ASM a non-exclusive, world-wide license to publish the Supplemental Material files. Please contact the corresponding author directly for reuse.
