# Supplementary material for: Inhibitory effect of natural flavone luteolin on Streptococcus mutans biofilm formation
Source: Microbiol Spectr. 2023 Sep 21;11(5):e05223-22. doi: 10.1128/spectrum.05223-22 (PMC10581090; doi:10.1128/spectrum.05223-22)
Supplement: Supplemental figures — Fig. S1 to S9. [file spectrum.05223-22-s0001.docx]

**Supplementary Materials for Manuscript Entitled**

**Inhibitory effect of natural flavone luteolin on *Streptococcus mutans* biofilm formation**

Lucille Rudin^1^, Noelle Roth^1^, Julien Kneubühler^1^, Badri Nath Dubey^3^**,** Michael M. Bornstein^1,2^ and Viktoriya Shyp^1,2, *^

^1^Department Research, University Center for Dental Medicine Basel UZB, University of Basel, Basel, Switzerland.

^2^Department of Oral Health & Medicine, University Center for Dental Medicine Basel UZB, University of Basel, Basel, Switzerland.

^3^CSSB Centre for Structural Systems Biology Deutsches Elektronen-Synchrotron DESY, Notkestr. 85, 22607 Hamburg, Germany

*corresponding author: viktoriya.shyp@unibas.ch

**SUPPLEMENTAL FIGURES
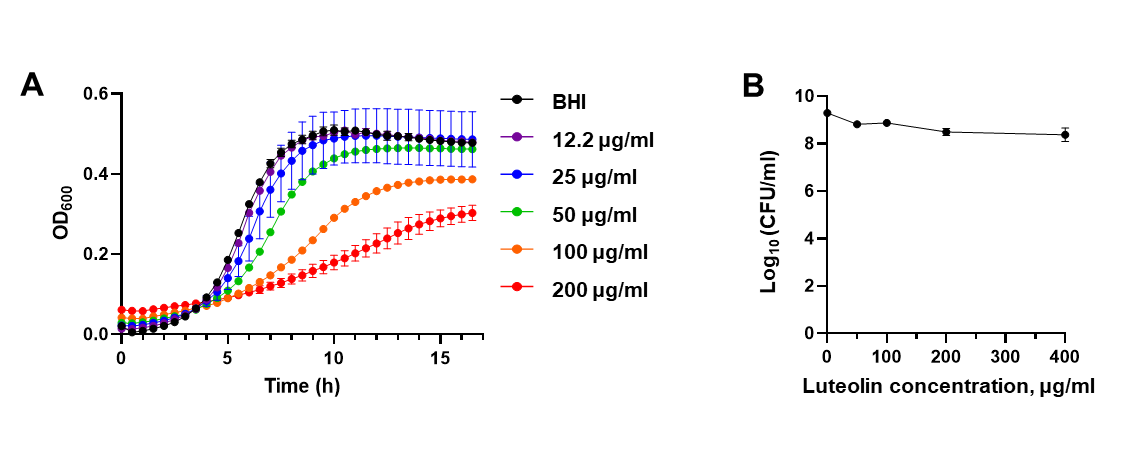
**

**Figure S1. Antibacterial effect of luteolin on *S. mutans*.** Luteolin effect on *S. mutans* planktonic growth (A) and bacterial cell viability in BHI as measured by CFU assay (B). Bacterial growth was monitored by measuring optical density at 600 nm. For CFU calculation, serial dilutions of the bacterial cultures after treatment with luteolin were plated on BHI agar plates and incubated 2 days aerobically.

**
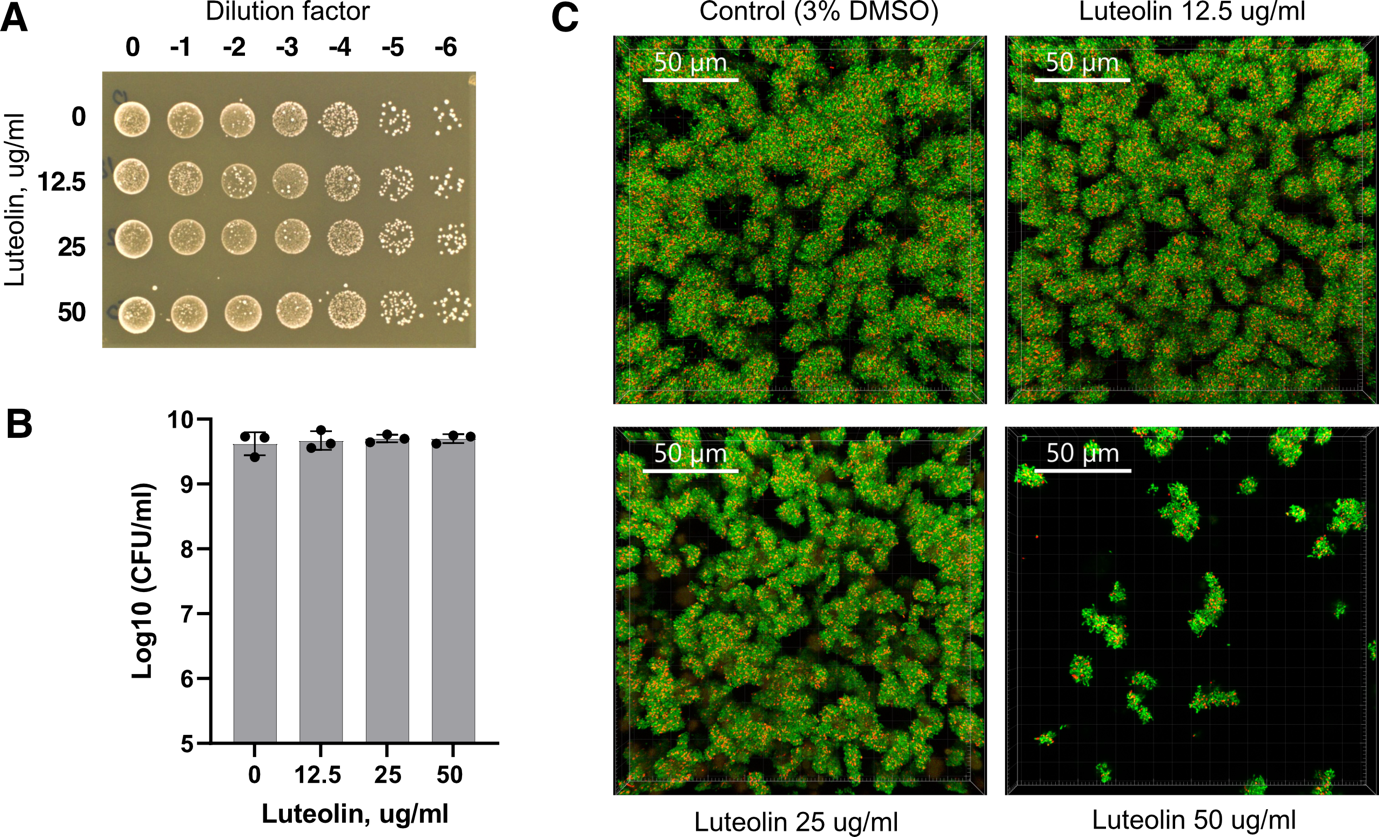
**

**Figure S2. Antibacterial effect of luteolin on *S. mutans* in biofilm.** A) Spot assay to assess cell viability in control and luteolin-treated biofilm. After 24 h of incubation in biofilm, all bacteria were resuspended in 200 ul of sterile PBS, serially diluted 10-fold (10^0^ – 10^-6^) and spotted on BHI agar medium. B) To determine CFU in control and treated biofilm, 100 ul of 10^-6^ dilution were plated on BHI agar and incubated for 48 h at 37°C. (C) Representative images of live/dead-stained 24-h biofilms treated with luteolin as compared to the solvent control. Live bacteria are shown in green and dead (PI-stained) in red. Image reconstructions were performed with Imaris 9.0.0.


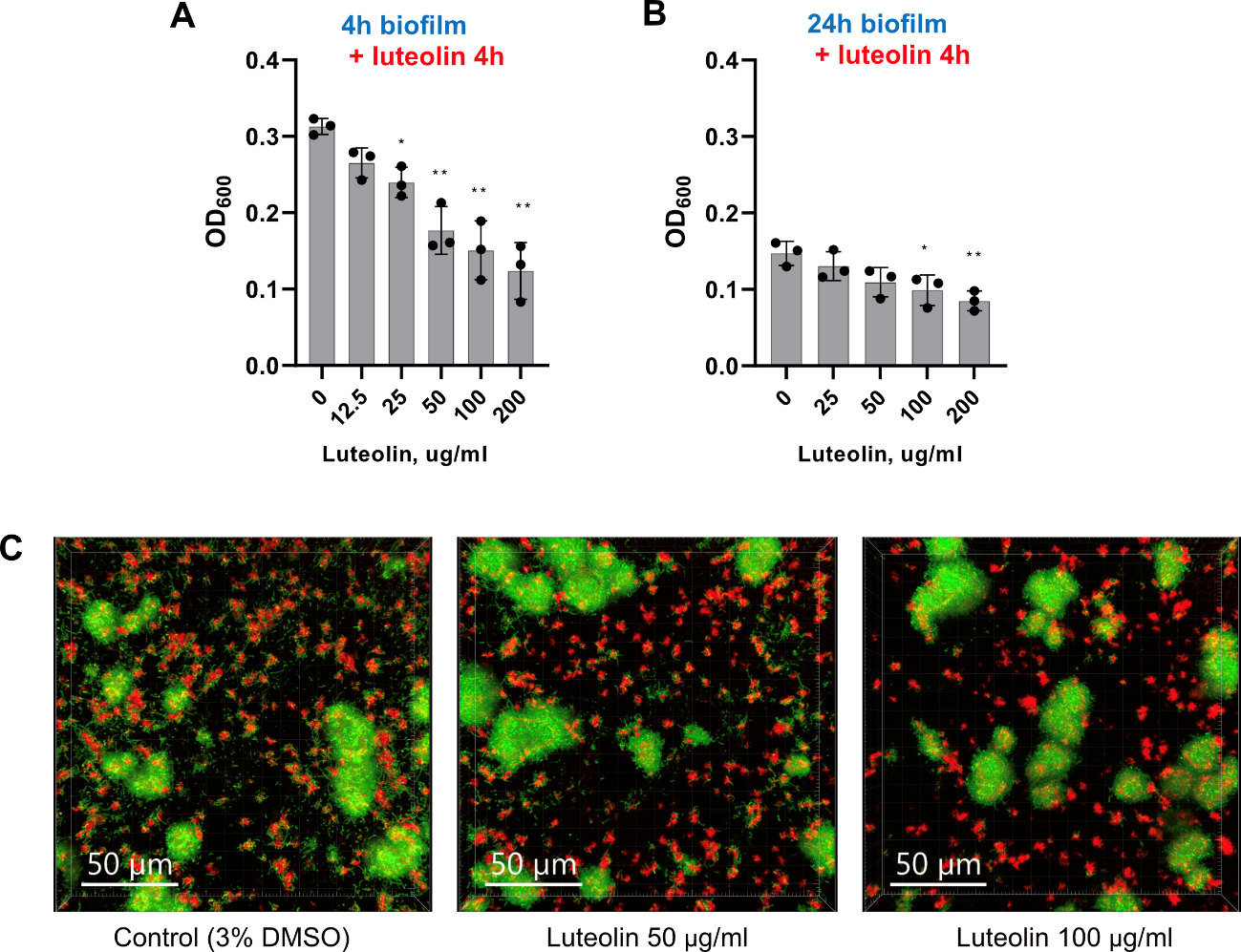


**Figure S3. Effect of luteolin on preformed biofilm dispersal of *S. mutans***. 4- (A) and 24-h (B) *S. mutans* biofilms were treated with luteolin for additional 4 h. Cell released into the media were measured at OD_600_. (C) Representative CLSM images of the 4-h preformed biofilm treated with luteolin. Bacteria were stained with SYTO9 (in green), EPS were labeled with Alexa Fluor 647 (in red).

**
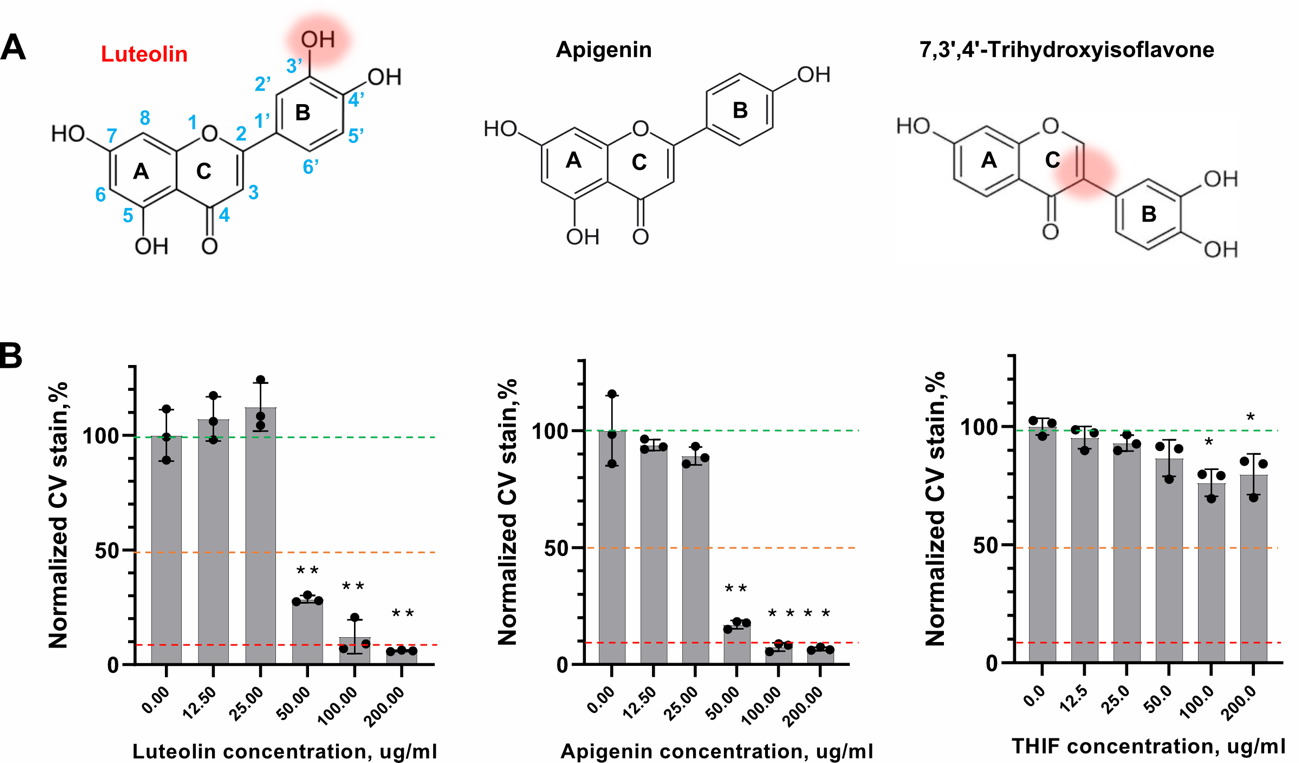
**

**Figure S4. Comparison of antibiofilm effect of luteolin with apigenin and structurally similar isoflavone. (A)** Chemical structure of tested flavonoids. **(B)** The effect of flavonoids on 24-h biofilm of *S. mutans*. Biofilm mass was quantified by CV staining and measuring absorbance at 595 nm. Values were normalized to non-treated cells (100%, green dashed line). Orange and red dashed lines indicate the reduction in biofilm mass up to 50% and 90%, respectively. Bars represent the mean of three biological replicates. Error bars show standard deviation.


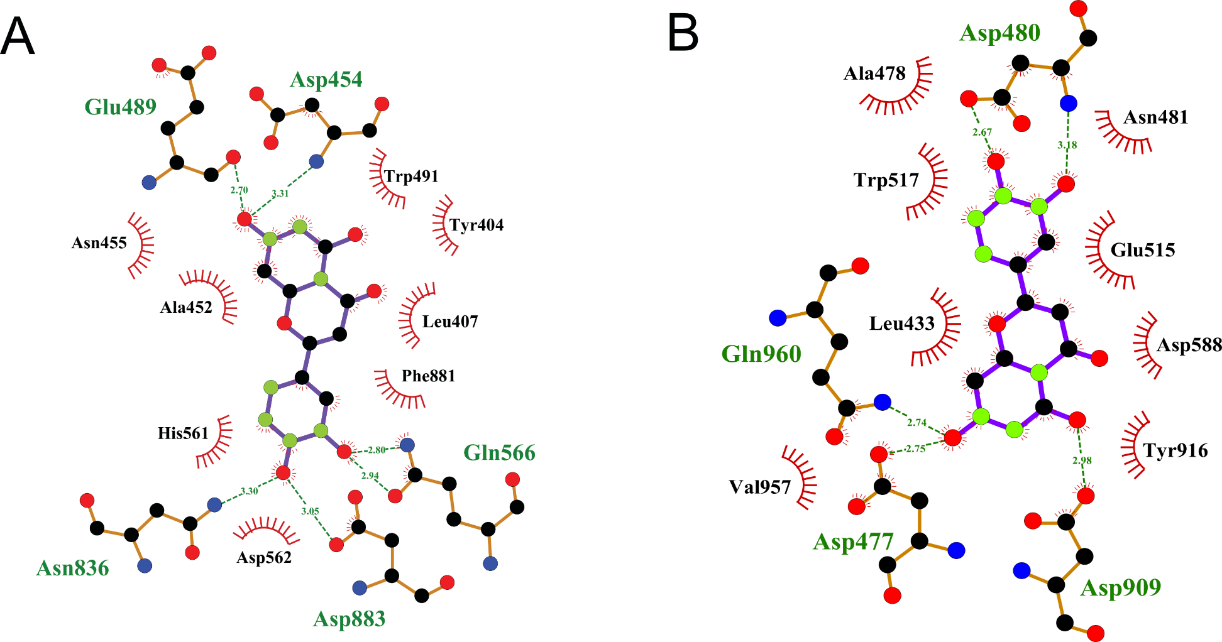


**Figure S5. LIGPLOTs illustrating the interaction of luteolin with the active site of GtfB (A) and GtfC (B).** The amino acid residues of enzymes involved in hydrophobic interactions are depicted as crescents with bristles, where the orientation of bristles reflects corresponding direction of contacts with luteolin atoms with bristles. The polar atoms, oxygen and nitrogen, are shown in red and blue, respectively. Hydrogen bonds are indicated by green dashes.


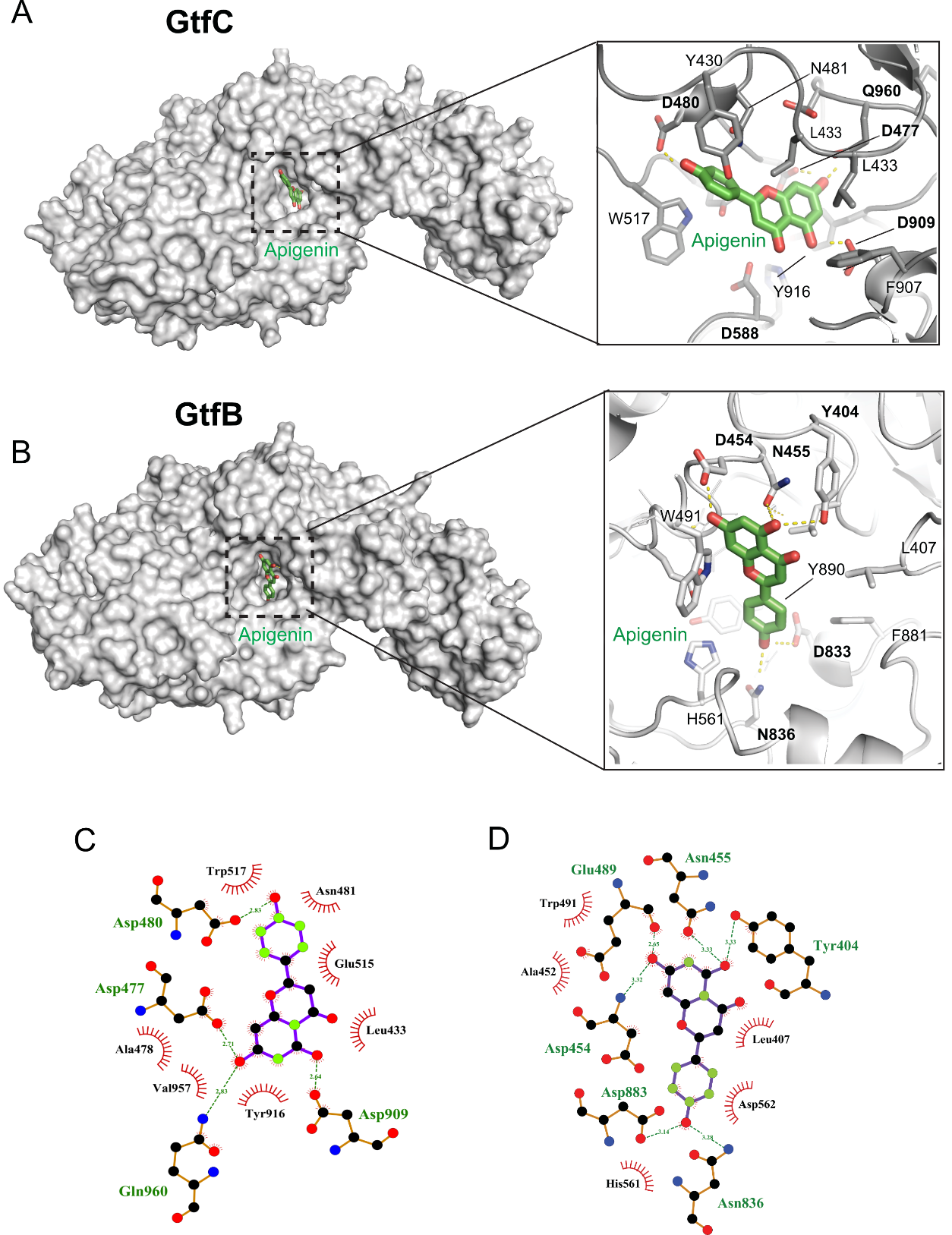


**Figure S6**. A-B) Left: surface representation of apigenin (shown in green stick) docked with GtfC and GtfB. Right: best-docked pose of apigenin in the active sites of S. mutans GtfC (PDB code3AIE) and GtfB (PDB code 8FK4). Residues involved in hydrogen bonding are labelled as bold. C-D) LIGPLOTs illustrating the interaction of apigenin with the active site of GtfC (C) and GtfB (D). The amino acid residues of the enzymes involved in hydrophobic interactions are depicted as crescents with bristles, where the orientation of bristles reflects corresponding direction of contacts with apigenin atoms with bristles. The polar atoms, oxygen and nitrogen, are shown in red and blue, respectively. Hydrogen bonds are indicated by green dashes.


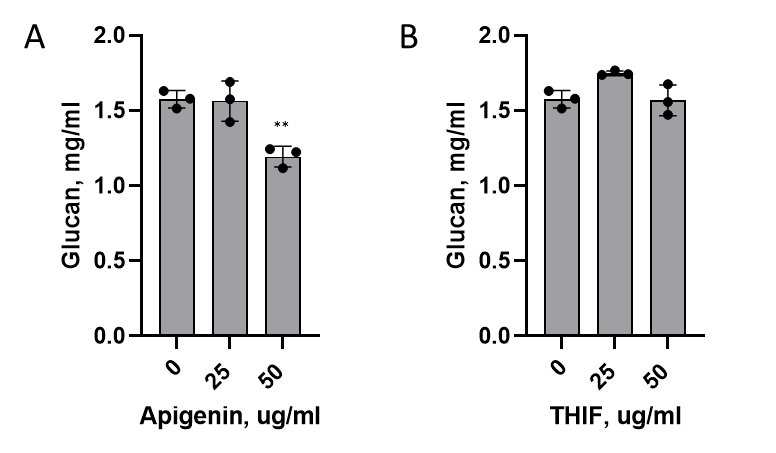


**Figure S7. Apigenin (A) and THIF (B) effect on biofilm-associated water-insoluble glucans as measured by anthrone method.**


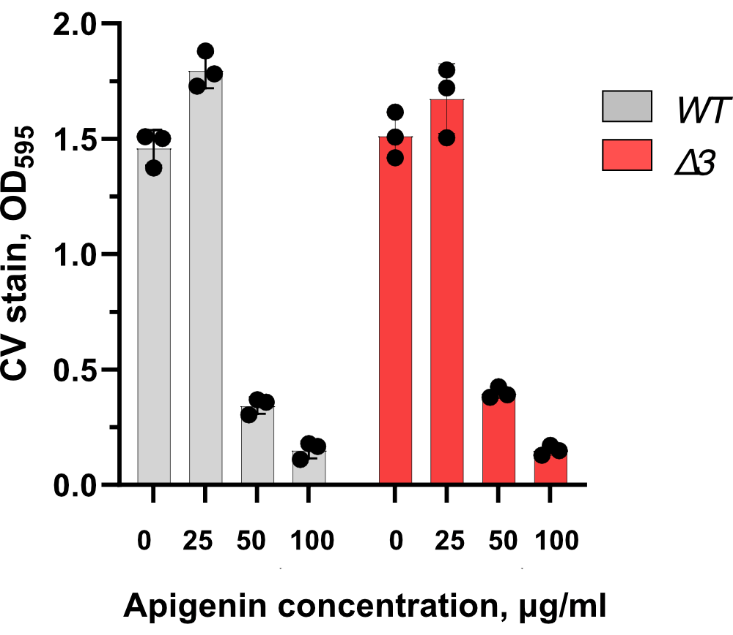


**Figure S8.** The effect of apigenin on 24-h biofilms of S. mutans wild type (in grey) and *Δ3* mutant (in red) as quantified by CV staining. Bars represent the mean of three biological replicates. Error bars show standard deviation.


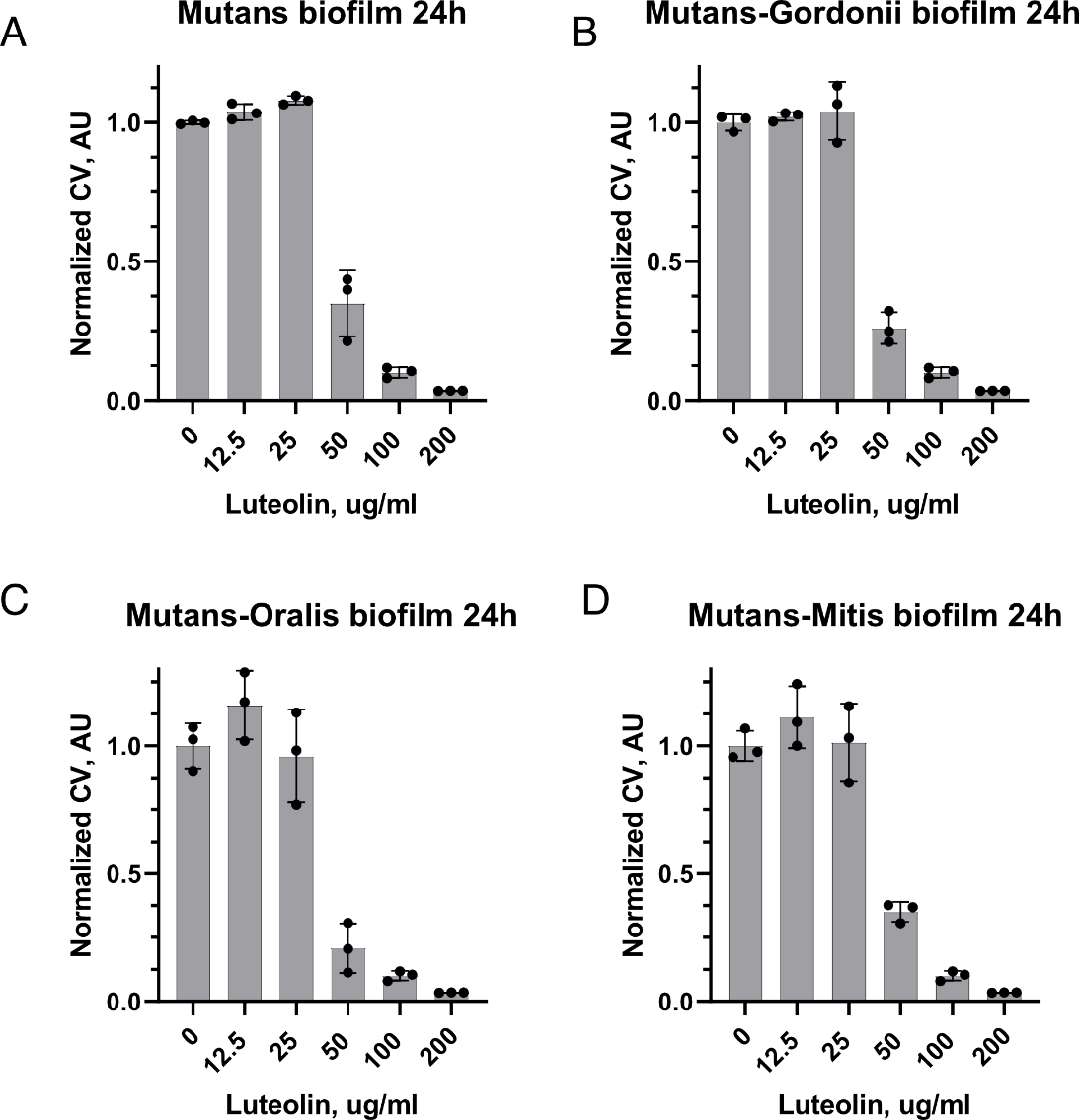


**Figure S9.** The effect of luteolin on 24 h old dual-species biofilm of *S. mutans* with oral commensal streptococci.
