# Supplementary material for: Inhibitory effect of natural flavone luteolin on Streptococcus mutans biofilm formation
Source: Microbiol Spectr. 2023 Sep 21;11(5):e05223-22. doi: 10.1128/spectrum.05223-22 (PMC10581090; doi:10.1128/spectrum.05223-22)
Supplement: Tables S1 and S2 — Primers used for mutant strain construction and for qRT-PCR. [file spectrum.05223-22-s0002.pdf]

Supplementary Materials for Manuscript Entitled

Inhibitory effect of natural flavone luteolin on *Streptococcus mutans* biofilm formation

Lucille Rudin[1], Noelle Roth[1], Julien Kneubühler[1], Michael M. Bornstein[1,2] and Viktoriya Shyp[1,2]

SUPPLEMENTAL TABLE 1. Primers used to construct Δ3 *S. mutans* strain.

Primers	Sequences
**043_IFDC2-forw**	CCGAGCAACAATAACACTCATAG
**044_IFDC2-rev**	GAAGCTGTCAGTAGTATACCTAATAA
**049_wapA-up-forw**	GTTATTGAAACTAATGGTGCCAGC
**050_wapA-up-rev-IFDC2**	ATGAGTGTTATTGTTGCTCGGAATAGAATTTTCTCCTTAGTTAATC
**051_wapA-dn-forw-IFDC2**	TAGGTATACTACTGACAGCTTCCATTGATTATGCTAATAAAACCGAG
**052_wapA-dn-rev**	CAGCATAACAGCAGTATCTTTCCA
**053_spaP-up-forw**	TGATGCTAAACTGTCTAGTCCGAC
**054_spaP-up-rev-IFDC**	ATGAGTGTTATTGTTGCTCGGAAATCCTCCAAATCTGAATAAAT
**055_spaP-dn-forw-IFDC2**	TAGGTATACTACTGACAGCTTCCAGCATAGATATTACATTAGAATTA
**056_spaP-dn-rev**	CATAACTTTCAGGGTCTTGCGGAA
**057_Smu_063-up-forw**	CTCACTTGAATTTTTAACTTCTAGC
**058_Smu_063-up-rev-IFDC2**	ATGAGTGTTATTGTTGCTCGGTATAGTTTCTAACCCTTCTAAAAAG
**059_Smu_063-dn-forw-IFDC2**	TAGGTATACTACTGACAGCTTCTAAGTTAATTTAAATTCTCTAAAA
**060_Smu_063-dn-rev**	CCAATTATCAGGTCAATGATTTGA
**032-spaP_up-rev**	TAATTCTAATGTAATATCTATGCTGAAATCCTCCAAATCTGAATAAATCTT
**033-spaP_dn-forw**	AAGATTTATTCAGATTTGGAGGATTTCAGCATAGATATTACATTAGAATTA
**038-wapA_up-rev**	CTCGGTTTTATTAGCATAATCAATGAATAGAATTTTCTCCTTAGTTAAT
**039-wapA_dn-forw**	ATTAACTAAGGAGAAAATTCTATTCATTGATTATGCTAATAAAACCGAG
**026-Smu_63c_up-rev**	ACTTTTTAGAAGGGTTAGAAACTATATAAGTTAATTTAAATTCTCTAAAA
**027-Smu_63c_dn-forw**	TTTTAGAGAATTTAAATTAACTTATATAGTTTCTAACCCTTCTAAAAAGT

SUPPLEMENTAL TABLE 2. Primers used for qRT-PCR.

Primers	Sequences
**090-16S-forw**	AGCGTTGTCCGGATTTATTG
**091-16S-rev**	CTACGCATTTCACCGCTACA
**087-spaP-forw**	GACTTTGGTAATGGTTATGCATC
**088-spaP-Rev**	TTTGTATCAGCCGGATCAAGT
**098-gbpC-F**	GGCGATCATGTGGAAAAAGT
**099-gbpC-R**	ATAATAAGCCGTCGCAGCAC
**081-gtfB-Forw**	CACTATCGGCGGTTACGAAT
**082-gtfB-Rev**	CAATTTGGAGCAAGTCAGCA
**083-gtfC-Forw**	GATGCTGCAAACTTCGAACA
**084-gtfC-rev**	TATTGACGCTGCGTTTCTTG
**085-gtfD-Forw**	TTGACGGTGTTCGTGTTGAT
**086-gtfD-rev**	AAAGCGATAGGCGCAGTTTA
**079-ftf-Forw**	AAATATGAAGGCGGCTACAAC
**080-ftf-Rev**	TTCACCAGTCTTAGCATCCTG
