# Supplementary material for: Inhibitory effect of natural flavone luteolin on Streptococcus mutans biofilm formation
Source: Microbiol Spectr. 2023 Sep 21;11(5):e05223-22. doi: 10.1128/spectrum.05223-22 (PMC10581090; doi:10.1128/spectrum.05223-22)
Supplement: Supplemental methods — Additional experimental details for S. mutans mutant strain construction, trasformation, and real-time PCR. [file spectrum.05223-22-s0003.pdf]

Supplementary Materials for Manuscript Entitled

Inhibitory effect of natural flavone luteolin on *Streptococcus mutans* biofilm formation

Lucille Rudin[1], Noelle Roth[1], Julien Kneubühler[1], Michael M. Bornstein[1,2] and Viktoriya Shyp[1,2]

SUPPLEMENTAL METHODS

Construction of Δ3 mutant. A triple mutant (Δ3) lacking P1 (SpaP), WapA, and Smu_63c amyloid proteins was generated using a markerless two-step integration/excision mutation strategy described previously (Merritt et al. 2007; Xie et al. 2011). The counterselectable resistance cassette IFDC2 encoding erythromycin resistance gene and mutated phenylalanine tRNA synthetase gene (provided by J. Merritt) was amplified and flanked by the sequences homologous the *spaP*, *wapA* or *smu_063* immediately upstream and downstream of the ORFs. The cassette was amplified using the primer pairs 043_IFDC2-forw/044-IFDC2-rev. The flanking regions for each separate gene deletion were amplified from the *S. mutans* genomic DNA using primer pairs 049_wapA-up-forw/050_wapA-up-rev-IFDC2 and 051_wapA-dn-forw-IFDC2/052_wapA-dn-rev, 053_spaP-up-forw/054_spaP-up-rev-IFDC2 and 055_spaP-dn-forw-IFDC2/056_spaP-dn-rev, and 057_Smu_063-up-forw/058_Smu_063-up-rev-IFDC2 and 059_Smu_063-dn-forw-IFDC2/060_Smu_063-dn-rev. Flanking fragments were assembled with IFDC2 cassette via OE-PCR with the primer pairs 049_wapA-up-forw/052_wapA-dn-rev, 053_spaP-up-forw/056_spaP-dn-rev, and 057_Smu_063-up-forw/060_Smu_063-dn-rev, respectively. Resulting amplicons were used for *S. mutans* cells transformation (see Appendix 2 for details) followed by the selection on BHI agar plates supplemented with 12.5 ug/ml erythromycin. Next, to remove the IFDC2 cassette and generate markerless deletion mutant, erythromycin resistant strains were transformed with the second construct, and selected on BHI agar plate supplemented with 0.4% (wt/vol) 4- chlorophenylalanine (4-CP). For the second construct, *spaP*, *wapA* or *smu_063* ORF flanking fragments were amplified from the genomic DNA with the primer pairs 053_spaP-up-forw/032-spaP_up-rev, 033-spaP_dn-forw/056_spaP-dn-rev; 049_wapA-up-forw/038-wapA_up-rev, 039-wapA_dn-forw/052_wapA-dn-rev; 057_Smu_063-up-forw/026-Smu_63c_up-rev, 027-Smu_63c_dn-forw/060_Smu_063-dn-rev, respectively and fragments were assembled with via OE-PCR. Resulting deletion have been confirmed by PCR and sequencing. Primers used for the mutagenesis are listed in Appendix Table 1.

Transformation via electroporation

To prepare electro-competent cells, overnight cultures of *S. mutans* were diluted 1:20 in BHI and grown at 37°C in 5% CO_2 to an OD_{600} of 0.2 - 0.3. Subsequently, 2 ml cultures were transferred on ice for 15 min and then centrifuged for 15 min at 4,000 r.p.m. The supernatant was discarded and the pellet was washed twice in ultrapure ice-cold water and once in 10% ice-cold glycerol. The final pellet was resuspended in 90 µl of 10% glycerol and incubated on ice for 20 min with 1 µg of purified DNA. The cells were electroporated with BTX electroporation system (voltage 1.25 kV) in 1mm gap cuvette (Lubio Science, Zürich, Switzerland) and then immediately transferred on ice. Cells were diluted in 1 ml BHI and recovered at 37 °C for 1h. After incubation, the cells were plated on selective agar plate and incubated at 37 °C for next 48 h. Transformants were subcultured, checked via PCR and verified by sequencing.

S. mutans gene expression analysis by real-time PCR

To determine the effect of luteolin on the gene expression levels in biofilm, *S. mutans* cells were cultured in 12-well plate for 24 hours with 25 µg/ml luteolin as described above for the biofilm assay. Culture without luteolin served as a control. After 24-hour incubation, supernatants were carefully removed from the wells, biofilms were scarped with sterile spatula and resuspended in 1 ml RNAprotect bacterial reagent (Qiagen). After incubation at RT for 5 min, pellets were collected by centrifugation for 10 min at 10 000 x g. Stabilized pellet was resuspended in 5 ml cold PBS and homogenized by sonication on ice to remove soluble polysaccharides. Three cycles of washing-ultrasonication were performed with 30s pulse. Cells were resuspended in buffer with 15mg/ml lysozyme and incubated for 15 min at RT. 1 ml of TRIzol reagent (Invitrogen) was added to the suspension, and then transferred to the 2-ml screw-cap tubes with 0.5 mm acid-washed glass beads. Cell were lyzed by vertexing for 10 min at the highest speed. Total RNA was extracted using chloroform following the extraction protocol from RNeasy kit (Qiagen) with on-column DNase digestion step. The cDNA was synthesized using GoScript™ Reverse Transcriptase system with random primers (Promega, Switzerland). To quantify the mRNA expression, qRT–PCR was performed using FastStart Universal SYBR Green Master (ROX) (Roche Diagnostics, Mannheim, Germany). The PCR was performed in CFX96 Real-Time System (BioRad) were in accordance to the manufacturer's recommendations for determining the threshold cycle values (Ct). The sequences of the primers used in this study are listed in Appendix Table 2. 16S rRNA was used as an internal control.

SUPPLEMENTAL REFERENCES

Merritt J, Tsang P, Zheng L, Shi W, Qi F. 2007. Construction of a counterselection-based in-frame deletion system for genetic studies of streptococcus mutans. Oral Microbiol Immunol. 22(2):95-102.

Xie Z, Okinaga T, Qi F, Zhang Z, Merritt J. 2011. Cloning-independent and counterselectable markerless mutagenesis system in streptococcus mutans. Appl Environ Microbiol. 77(22):8025-8033.
